# Single-Stranded Nucleic Acids Regulate TLR3/4/7 Activation through Interference with Clathrin-Mediated Endocytosis

**DOI:** 10.1038/s41598-018-33960-4

**Published:** 2018-10-26

**Authors:** Peter Järver, Aleksandra Dondalska, Candice Poux, AnnSofi Sandberg, Joseph Bergenstråhle, Annette E. Sköld, Nathalie Dereuddre-Bosquet, Fréderic Martinon, Sandra Pålsson, Eman Zaghloul, David Brodin, Birgitta Sander, Kim A. Lennox, Mark A. Behlke, Samir EL-Andaloussi, Janne Lehtiö, Joakim Lundeberg, Roger LeGrand, Anna-Lena Spetz

**Affiliations:** 1 Department of Molecular Biosciences, Wenner-Gren Institute, Stockholm University, 106 91 Stockholm, Sweden; 2 Cancer Proteomics Mass Spectrometry, Department of Oncology-Pathology, Science for Life Laboratory, Karolinska Institutet, 171 65 Stockholm, Sweden; 3 Department of Gene Technology, Science for Life Laboratory, Royal Institute of Technology, 171 65 Solna, Sweden; 4 CEA, -Université Paris Sud-Inserm U1184, IDMIT Department, Institut de Biologie Francois Jacob (IBFJ), 922 60 Fontenay-aux-Roses, France; 5 Clinical Research Center, Department of Laboratory Medicine, Karolinska Institutet, 141 86 Stockholm, Sweden; 6 Bioinformatics and Expression Analysis core facility, Department of Biosciences and Nutrition, Karolinska Institutet, 141 83 Stockholm, Sweden; 7 Division of Pathology, Department of Laboratory Medicine, Karolinska Institutet, 141 86 Stockholm, Sweden; 8 Integrated DNA Technologies Inc, Coralville, 52241 Iowa USA; 9 Department of Physiology, Anatomy and Genetics, University of Oxford, OX1 3PT Oxford, UK

## Abstract

Recognition of nucleic acids by endosomal Toll-like receptors (TLR) is essential to combat pathogens, but requires strict control to limit inflammatory responses. The mechanisms governing this tight regulation are unclear. We found that single-stranded oligonucleotides (ssON) inhibit endocytic pathways used by cargo destined for TLR3/4/7 signaling endosomes. Both ssDNA and ssRNA conferred the endocytic inhibition, it was concentration dependent, and required a certain ssON length. The ssON-mediated inhibition modulated signaling downstream of TLRs that localized within the affected endosomal pathway. We further show that injection of ssON dampens dsRNA-mediated inflammatory responses in the skin of non-human primates. These studies reveal a regulatory role for extracellular ssON in the endocytic uptake of TLR ligands and provide a mechanistic explanation of their immunomodulation. The identified ssON-mediated interference of endocytosis (SOMIE) is a regulatory process that temporarily dampens TLR3/4/7 signaling, thereby averting excessive immune responses.

## Introduction

Pathogenic infections and tissue damage that lead to the release of nucleic acids activate pattern recognition receptors (PRR), resulting in a rapid inflammatory response^1^. The nucleic acid sensing PRR include RIG-I like receptors (RIG-I, LGP2, DDX3 and MDA5), cytosolic DNA sensors, and a subgroup of TLRs consisting of TLR3, 7, 8, and 9, as well as murine TLR13^1^. TLRs are highly, but variably expressed in immune cells, endothelial cells, epithelial cells and keratinocytes^2^. TLR3, 7, 8, and 9 all primarily reside in the endosomes, in contrast to other nucleic acid sensors, which are cytosolic. TLRs are type I transmembrane receptors composed of three domains: an extracellular leucine-rich-repeat domain, a transmembrane domain and a cytoplasmic tail that contains a Toll-IL1R domain^3^.

The endosomal TLRs (3, 7, 8 and 9) become stimulated upon binding ligands derived from pathogenic (bacterial or viral) nucleic acid degradation products, triggering an immune response^4^. DsRNA is a ligand for TLR3, ssRNA is a ligand for TLR7 and TLR8, and ssDNA containing un-methylated CpG motifs is a TLR9 ligand^3^. TLR7 and TLR8 can also respond to the small molecule R848^5^. Binding of agonists to TLR7, 8 and 9 triggers a signaling cascade beginning with the recruitment of the adaptor myeloid differentiation primary response 88 (Myd88)^3^. Alternatively, TLR3 binding activates the TIR-domain containing adaptor protein inducing interferon beta (TRIF) pathway for induction of type I interferons and inflammatory cytokine genes. TLR4, which senses bacterial lipopolysaccharides (LPS), has two distinct pathways; one MyD88-dependent pathway that signals from the plasma membrane, and one TRIF dependent pathway that is reliant on clathrin-mediated endocytosis (CME)^6–9^.

Recognition of microbial nucleic acids by endosomal or cytosolic PRR constitutes a key component in the innate immune system to combat viral infections. However, the limited structural differences in host and viral nucleic acids pose a clear challenge to enable discrimination between danger (i.e. infection and sterile tissue damage) and normal physiological cellular turnover^4,10^. During viral infections, viral dsRNA accumulates and triggers an innate immune response by activating TLR3. Moreover, endogenous nucleic acids can also trigger TLR3-dependent immune responses contributing to inflammatory pathologies and autoimmunity^11,12^. Therefore, it seems plausible that rigorous control prevents activation of endosomal TLRs by host nucleic acids. However, there is a lack in our understanding of such regulatory mechanisms, which set the threshold to restrict endosomal TLR activation. Self-nucleic acids released upon cell death are accessible to degradation by extracellular nucleases, whereas foreign nucleic acids are typically encapsulated by the bacterial cell wall or in viral particles and thus protected^4^. Endogenous nucleases can degrade self-nucleic acids before internalization into TLR signaling endosomes, mitigating the autoimmune potential. Mutations resulting in decreased activity of DNases and increased activation of endosomal TLRs have indeed been linked to several autoimmune diseases^4,10^. Further understanding of how to limit nucleic acid recognition by TLRs may have direct relevance to pathologies linked to unrestricted nucleic acid sensing, and may provide insights into potential therapeutic interventions.

SsON used in clinical studies, such as CpG adjuvants or anti-sense therapies, are internalized by endocytosis and then traffic through multiple membrane-bound intracellular compartments^13^. Synthetic ssDNA molecules with immunosuppressive functions are being studied in pre-clinical models; they vary in size, sequence and nucleotide backbone, but there is not yet full understanding on their mechanism of action^14^.

Although the cargoes for different endocytic pathways are well characterized, the regulation of their internalization is less clear^15^. In the present study, we have assessed whether extracellular ssON can modulate CME and macropinocytosis (MPC). CME is responsible for receptor-mediated endocytosis of ligands such as low-density lipoprotein (LDL), Transferrin (TF), and dsRNA and its analogue polyinosinic-polycytidylic acid (pI:C)^15,16^. MPC occurs from highly ruffled regions of the plasma membrane, and uptake indicators include fluid phase markers such as dextran^15^.

We previously showed that a 35mer CpG ssON could inhibit TLR3 signaling in primary human monocyte derived cells (moDC) that express TLR3/4/8, but lack TLR7/9^17^. In the present study, a panel of ssON was synthesized to identify the requirements for the inhibition of dsRNA-mediated activation (Table S1). We discovered that ssON not only inhibited TLR3 activation, but also inhibited the activation of TLR7 in peripheral blood mononuclear cells (PBMC). Further, we show that ssON modulated TLR4 activation that was dependent on endosomal uptake, while leaving signaling from the plasma membrane unaffected. We provide evidence that certain ssON temporarily shut down CME without causing major harm to the cell, as shown by viability assay, cytokine production, RNAseq and whole cell proteomic analyses. Finally, we demonstrate that ssON can shape immune responses induced by dsRNA locally in the skin of macaques. These findings show that certain ssON can inhibit CME, providing a mechanism to temporarily shut down the uptake of cargo to endosomes with subsequent dampening of inflammatory signatures.

## Results

### SsON inhibit endocytosis of pI:C, TF and LDL but spare uptake of dextran

**Figure 1:**
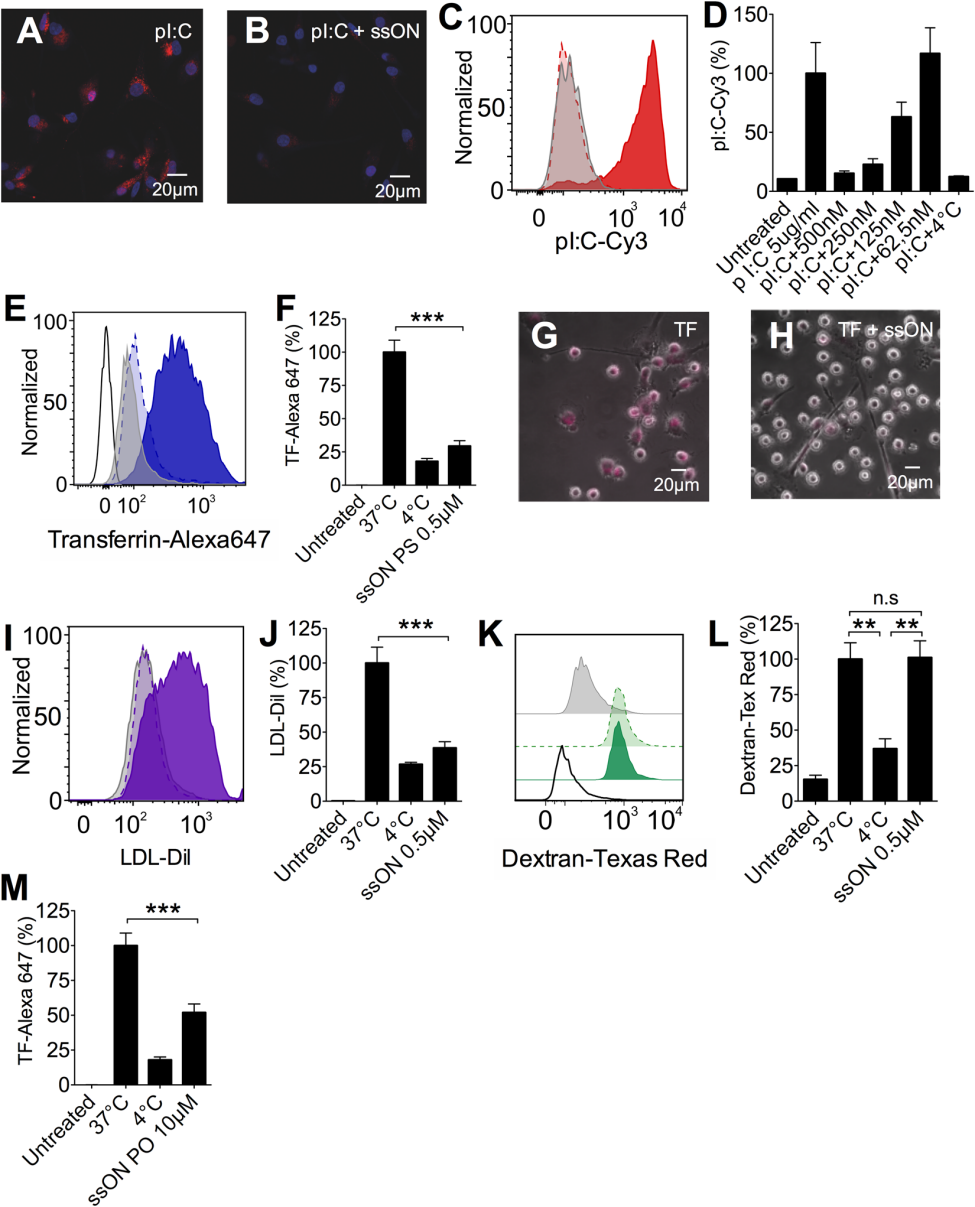
SsON inhibit CME and spare MPC in human moDC. Human moDC were treated for 45 min at +37 °C or +4 °C with endocytic uptake markers (pI:C-Cy3, TF-Alexa647, LDL-Dil, and Dextran-Texas Red) with or without addition of ssON. Flow cytometry histograms show representative data from at least three donors, in separate experiments. Red, blue, purple or green histograms are without ssON. Lighter colors with dashed lines depict the addition of ssON. Grey display background (fluorescent signal at +4 °C). (**A**,**B**) Confocal microscopy of pI:C uptake in the absence (**A**) or presence of 0.5 µM ssON 35 PS at + 37 °C (**B**). (**C**) 0.5 µM ssON 35 PS inhibited uptake of pI:C. (**D**) The inhibition of pI:C uptake was dependent on ssON 35 PS concentration. (**E**) Flow cytometry analysis of TF uptake in the presence of 0.5 µM ssON 35 PS at + 37 °C or +4 °C. (**F**) Quantification of TF uptake in the presence of 0.5 µM ssON 35 PS at +37 °C or +4 °C. (**G**,**H**) Wide-field microscopy of TF uptake in the absence (**G**) or presence of 0.5 µM ssON 35 PS at +37 °C (**H**). (**I**) Flow cytometry analysis of LDL uptake in the presence of 0.5 µM ssON 35 PS at +37 °C or +4 °C. (**J**) Quantification of LDL uptake in the presence of 0.5 µM ssON 35 PS at +37 °C or +4 °C. (**K**) Flow cytometry analysis of Dextran uptake in the presence of 0.5 µM ssON 35 PS at +37 °C or +4 °C. (**L**) Quantification of Dextran uptake in the presence of 0.5 µM ssON 35 PS at +37 °C or +4 °C. (**M**) 10 µM ssON 35 PO partly inhibited uptake of CME (TF). All data are from at least three donors in duplicate. Error bars are given in SEM. Non-parametric Mann-Whitney test was used to compare the data. P-value: not significant (n.s) P > 0.05; *P ≤ 0.05; **P ≤ 0.01; ***P ≤ 0.001.

It was previously reported that ssON can inhibit cytokine production after pI:C-induced TLR3 signaling^17,18^. However, the mechanism governing this inhibition is not clear. Since the synthetic dsRNA ligand pI:C is taken up via CME^16^, we assessed whether extracellular ssON have the ability to modulate endocytic pathways. Immature moDC were treated for 45 min with fluorescently labelled pI:C, with or without the addition of ssON (Table S1). Confocal microscopy showed uptake of pI:C into moDC and that this uptake was efficiently blocked by the addition of phosphorothioate modified ssON (ssON 35 PS) (Fig. 1A,B). Flow cytometry allowed for quantification of the uptake and showed that the inhibition was concentration dependent (Fig. 1C,D). To gain insight as to whether the inhibition of uptake was specific for pI:C or whether other clathrin-dependent or independent endocytic pathways were affected, moDC were treated with TF, LDL or dextran. At +37 °C, all tested ligands were readily taken up by moDC within 45 min (Fig. 1E–L). We found significant increase in the fluorescent signal after incubation at +37 °C compared with +4 °C, confirming that the ligands were internalized versus binding to the cell surface. The fluorescent signal for TF was fully blocked by the addition of 0.5 µM ssON 35 PS, as measured by flow cytometry and confirmed by microscopy (Fig. 1E–H). Similarly, the cellular uptake of LDL was also completely inhibited by ssON 35 PS (Fig. 1I,J). The introduction of PS stabilizations was not a requirement for the effect, as shown by reduced uptake of TF with the addition of 10 µM of an unmodified phosphodiester ssON (ssON 35 PO) (Table S1, Fig. 1M). MPC remained unaffected by the treatment with ssON 35 PS, as demonstrated by the same uptake of dextran at +37 °C with or without the addition of 0.5 µM ssON 35 PS (Fig. 1K,L). Altogether, these findings show that ssON can significantly inhibit clathrin-mediated endocytosis of pI:C, TF and LDL, but leave MPC rates unaffected, suggesting a regulatory role for ssON in the uptake of cargo into endosomes.

### SsON temporarily inhibit TLR3 activation

**Figure 2:**
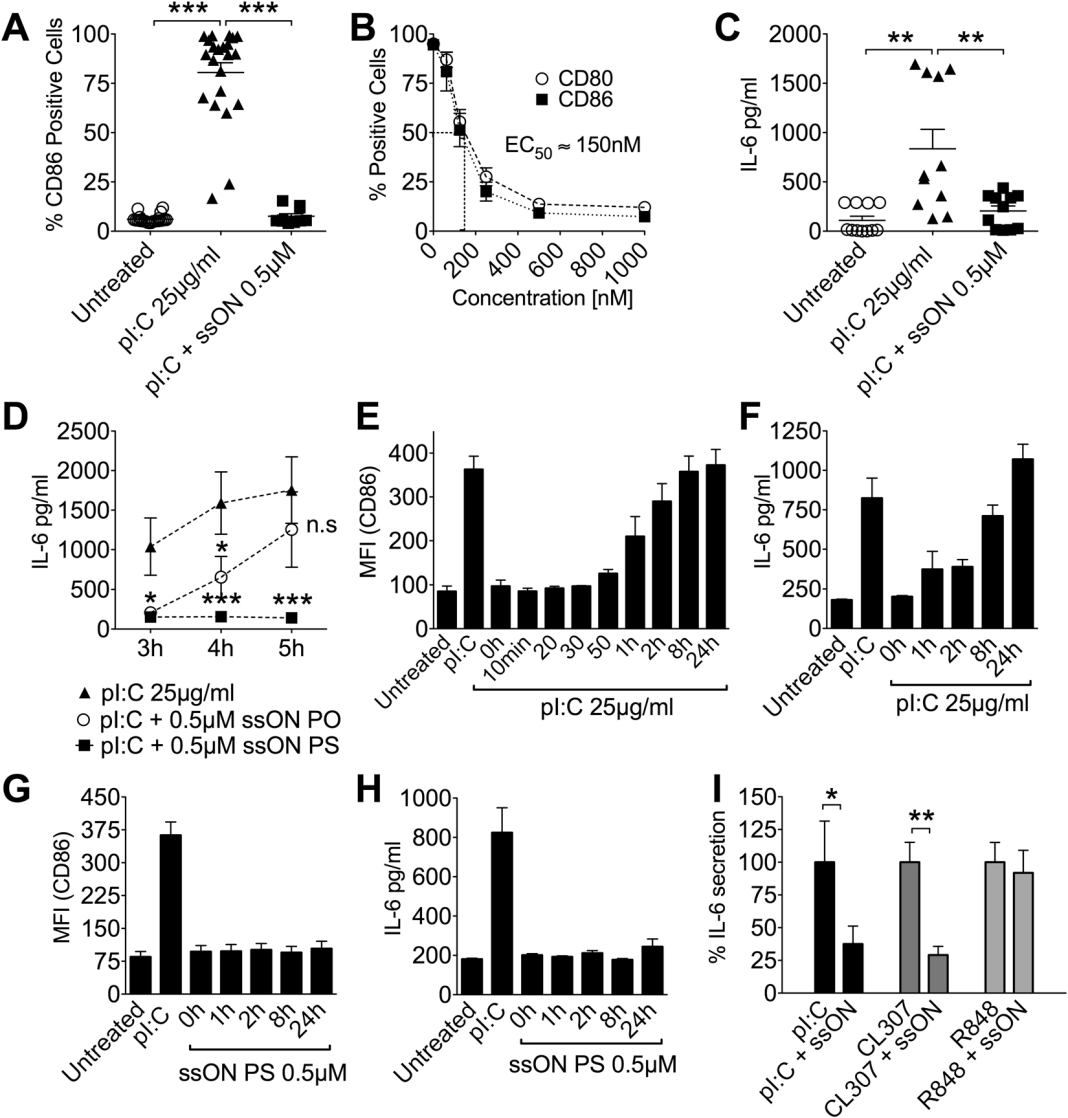
SsON inhibit TLR3 and TLR7 activation. (**A**) The frequency of moDC expressing CD86 was determined by flow cytometry. 0.5 µM ssON 35 PS completely inhibited pI:C-induced CD86 expression (48 h). Results from individual donors are shown. (**B**) The ssON 35 PS-mediated inhibition of CD86 and CD80 expression in pI:C exposed moDC was concentration dependent (48 h), EC_50_ between 100–200 nM). (**C**) SsON 35 PS completely inhibited pI:C-induced IL-6 secretion from moDC (48 h). ELISA results from individual donors are shown. (**D**) PS modification was not essential for the inhibitory effect. IL-6 released from moDC was quantified by ELISA. (**E**,**F**) CD86 expression and IL-6 secretion from moDC exposed to pI:C and subsequently challenged with ssON 35 PS at time-points from 5 min to 24 h. (**G**,**H**) MoDC treated in the opposite way, first ssON 35 PS and subsequently challenged with pI:C. (**I**) SsON mediated inhibition of IL-6 secretion in PBMC (24 h) was limited to agonists that are taken up by endocytosis (pI:C and CL307). Unless otherwise stated, all data are from at least three donors in duplicate. Error bars are given in SEM. Non-parametric Mann-Whitney test was used to compare the data. P-value: not significant (n.s) P > 0.05; *P ≤ 0.05; **P ≤ 0.01; ***P ≤ 0.001. See also Fig. S1.

We next investigated the functional kinetics of blocking endosomal uptake of the TLR3 ligand pI:C. PI:C-dependent moDC maturation was monitored 48 h post treatment by measuring the expression of co-stimulatory molecules in CD1a positive cells, and secretion of IL-6 24 h post treatment. SsON 35 PS significantly blocked pI:C mediated induction of CD86 and CD80, in a concentration dependent manner with an EC_50_ between 100–200 nM (Fig. 2A,B). Next we detected significant inhibition of pI:C-mediated IL-6 secretion from moDC and inhibition of TLR3 activation in TLR3 transfected HEK^blue^ cells by ssON 35 PS (Figs 2C and S1A). As with the uptake of TF, the inhibition of IL-6 was enhanced by, but not dependent on, stability modifications of the ssON (Fig. 2D). The ssON with a natural PO backbone is susceptible to nuclease degradation, while ssON with a stabilized PS backbone is more resistant^19^. At early time points the ssON with a PO backbone had similar efficacy as ssON 35 PS and the effect remained for 4 h, but diminished completely after 5 h (Fig. 2D). We infer from these results that native ssON with a PO backbone has the capacity to temporarily shut down the uptake of cargo into TLR3 signaling endosomes, but after nuclease degradation of the ssON, moDC regain capacity to respond to TLR3 stimulation. We could not detect ssON molecules in the cell culture media at the used working concentrations (0.5 µM), neither at time 0, nor at given time points (3–5 h). To verify that the ssON 35 PS is more stable than the PO ssON, we conducted a side-by-side nuclease degradation assay of ssON 35 PO and ssON 35 PS at a higher concentration (10 µM). Indeed, the ssON 35 PS oligonucleotide was stable for at least 24 h, while the native ssON 35 PO oligonucleotide was degraded within hours (Fig. S1B).

We studied whether transfection of pI:C is affected by the presence of ssON 35 PS. The lipid-based cationic transfection agent (LyoVec) aids delivery across the plasma membrane, and enables recognition of pI:C by cytosolic PRR such as RIG-I and MDA-5^20,21^. Initial uptake (45 min) of transfected pI:C was effectively blocked by 0.5 µM ssON 35 PS, as shown by confocal microscopy and quantified by flow cytometry (Fig. S2A–D). This is not unexpected because previous studies show that cationic lipid-based transfection agents employ endocytic pathways to enter the cell^22^. However, IL-6 secretion was only slightly reduced (not statistically significant) 24 h post treatment with pI:C/LyoVec in the presence of ssON 35 PS (Fig. S2E). We next treated the cells with 10 µM chloroquine, which prevents endosomal TLR3 activation by pI:C^23^ but allows for activation of cytosolic PRR. We found that chloroquine effectively blocked non-transfected pI:C-mediated induction of IL-6. However, IL-6 secretion was only slightly reduced after treatment with pI:C/LyoVec in the presence of chloroquine, similar to the reduction measured in the presence of ssON 35 PS (Fig. S2E). This indicates that although ssON 35 PS is able to inhibit initial uptake of pI:C/LyoVec, the transfected pI:C will eventually reach the cytosol and activate receptors such as RIG-I and MDA-5.

Previous studies show that it takes 35–60 min for moDC to endocytose extracellular ligands and engage receptor signaling^24^. To further analyse the kinetics involved in the ssON-mediated inhibition of pI:C uptake, moDC were exposed to TLR3 agonist pI:C and subsequently pulsed with ssON 35 PS over a period of time ranging from 5 min to 24 h. Expression of co-stimulatory molecule CD86 and secretion of IL-6 were measured to reveal pI:C-induced moDC maturation. Exposure to pI:C followed by pulsing with ssON 35 PS showed that the cells remained susceptible to inhibition for up to 50 min. However, after 50 min of incubation with pI:C, the moDC were not able to inhibit pI:C-induced activation (Fig. 2E,F). MoDC treated in the reverse fashion, with the stabilized ssON 35 PS first and then pulsed with pI:C, showed no sign of maturation or cytokine production for up to 24 h post initial treatment (Fig. 2G,H). These data show that the time frame during which ssON have the capacity to influence TLR3 activation coincides with the time that it takes (35–60 min) for moDC to endocytose and respond to extracellular compounds^24^. In addition, these data show that ssON would not inhibit already ongoing TLR3 activation in moDC and that the addition of ssON did not impair their capacity to express CD86 or produce IL-6.

PI:C can induce apoptosis in human cells^25^. To assess if ssON can prevent this, moDC were treated with pI:C with or without the addition of ssON 35 PS and cell death was measured. Cells treated with only pI:C had a reduced viability by approximately 20%. The frequency of cell death was reduced back to the level of untreated cells by the addition of 0.5 µM ssON 35 PS (Fig. S1C). No toxicity was observed in moDC exposed to 10 µM ssON 35 PS for 3 or 24 h (Fig. S1D,E) Altogether, these data show that ssON 35 PS was effective in preventing TLR3 activation, but could not inhibit ongoing signaling cascades, which is in accordance with the inhibition of pI:C uptake.

### Inhibition of endocytosis by ssON abolishes both TLR3 and TLR7 activation

As ssON-sensing TLRs are dependent on endocytosis for uptake of nucleic acids, and signaling occurs from endosomes^26^, we next assessed whether ssON have the ability to block TLR-mediated IL-6 production using the membrane permeable TLR7/8 agonist R848, which is not dependent on endocytosis to initiate signaling^5^. Alternatively, CL307 was used, which is a TLR7 agonist coupled to spermine and dependent on endocytosis for its cellular uptake^27^. Since moDC lack expression of ssRNA-sensing TLR7, these experiments were performed in PBMC. SsON 35 PS significantly reduced the secretion of IL-6 from PBMC treated with pI:C and CL307, but not in cells treated with R848 (Fig. 2I). These data show that ssON 35 PS have the ability to inhibit TLR3 and TLR7 activation mediated by endocytosis-dependent ligands.

### Partial ssON-mediated inhibition of TLR4 activation

**Figure 3:**
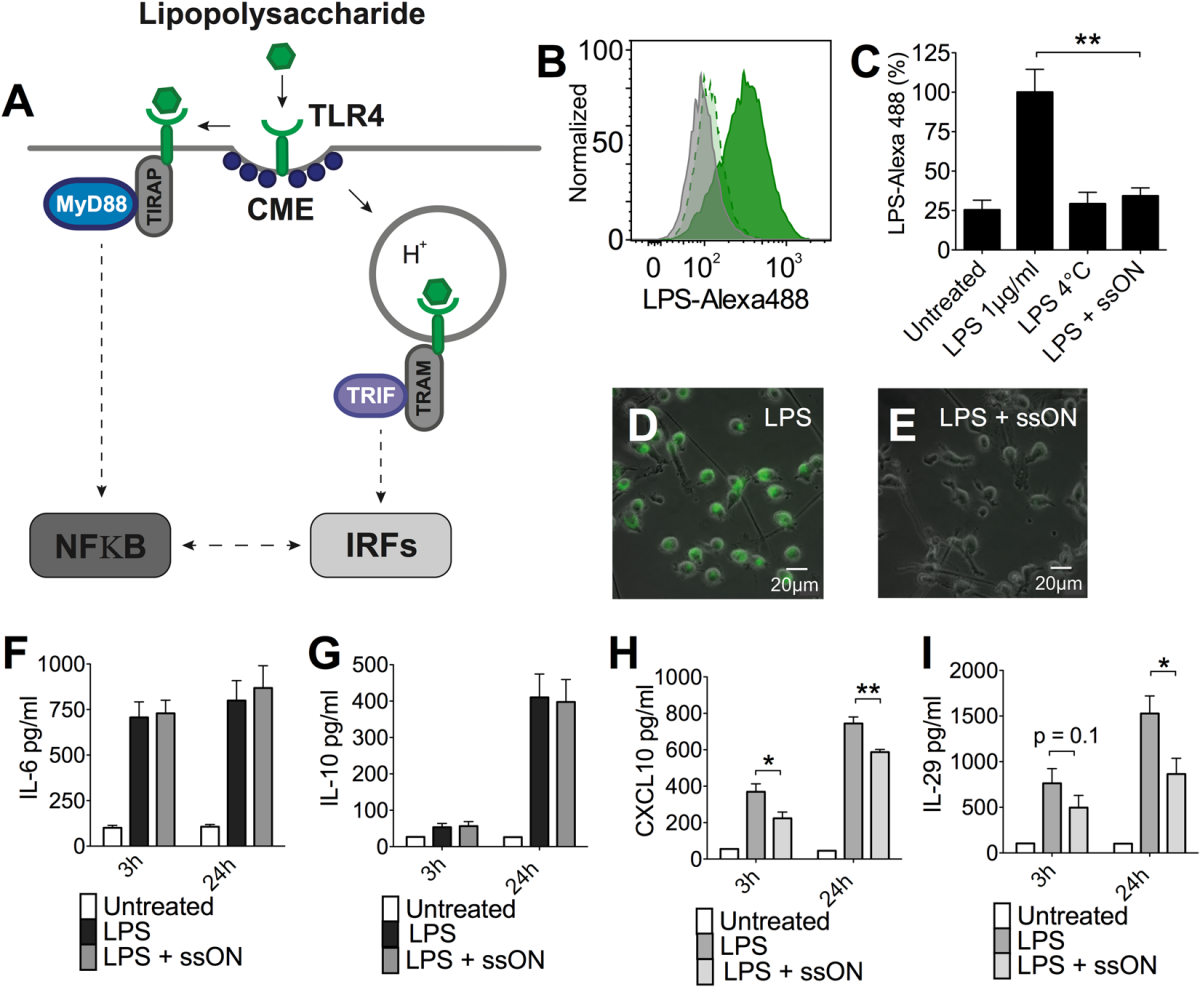
Partial inhibition of TLR4 activation in moDC exposed to ssON 35 PS. (**A**) Schematic view of TLR4-mediated cell signaling. Human moDC were exposed to the TLR4 agonist LPS with or without ssON 35 PS. (**B**) 0.5 µM ssON 35 PS inhibited uptake of fluorescently labelled LPS-Alexa488. Histograms show representative data from at least three donors. Dark green histogram is without ssON. Lighter color with a dashed lines depicts the addition of ssON. Grey displays background (fluorescent signal at +4 °C). (**C**) Quantitative analysis of LPS uptake measured by flow cytometry in the presence of 0.5 µM ssON 35 PS at +37 °C or +4 °C. (**D**) Wide-field microscopy of uptake of fluorescent LPS-Alexa488 in moDC (**E**) in the presence of 0.5 µM ssON 35 PS. (**F**,**G**) 0.5 µM ssON 35 PS had no influence on secretion of cytokines/chemokines that are downstream of NF-κB-mediated signaling pathways (IL-6 and IL-10). (**H**,**I**) 0.5 µM ssON 35 PS reduced secretion of cytokines/chemokines that are downstream of TRIF mediated signaling pathways (IL-29 and CXCL10). All data are from at least three donors in duplicate. Error bars are given in SEM. Non-parametric Mann-Whitney test was used to compare the data. P-value: not significant (n.s) P > 0.05; *P ≤ 0.05; **P ≤ 0.01; ***P ≤ 0.001.

TLR4 responds to LPS stimulation and has been reported to engage two distinct signaling pathways in DCs but not in macrophages^7–9^. One pathway is mediated through MyD88 and occurs at the plasma membrane leading to extensive NF-κB activation. The second pathway is mediated through TRIF, stimulating a strong type I interferon response. The TRIF-mediated signaling occurs from endosomes dependent on CME (Fig. 3A)^28^. As we found that ssON inhibited uptake of LPS, we reasoned that the addition of ssON 35 PS in combination with a TLR4 agonist should primarily inhibit the interferon response in moDC, (Fig. 3B–E). When moDC were simultaneously exposed to LPS and ssON 35 PS, the secretion of IL-6 and IL-10, which are primarily downstream of the MyD88 dependent pathway, remained unaffected (Fig. 3F,G). However, we observed significant inhibition of TRIF-dependent CXCL10^29^ and IL-29 secretion^30^ after stimulation with LPS in the presence of ssON 35 PS (Fig. 3H,I). These data imply that ssON 35 PS selectively affect the LPS-induced TLR4 signaling pathway that requires endocytosis.

### Length requirement of at least 25 bases for ssON´s capacity to inhibit TLR3 activation

**Figure 4:**
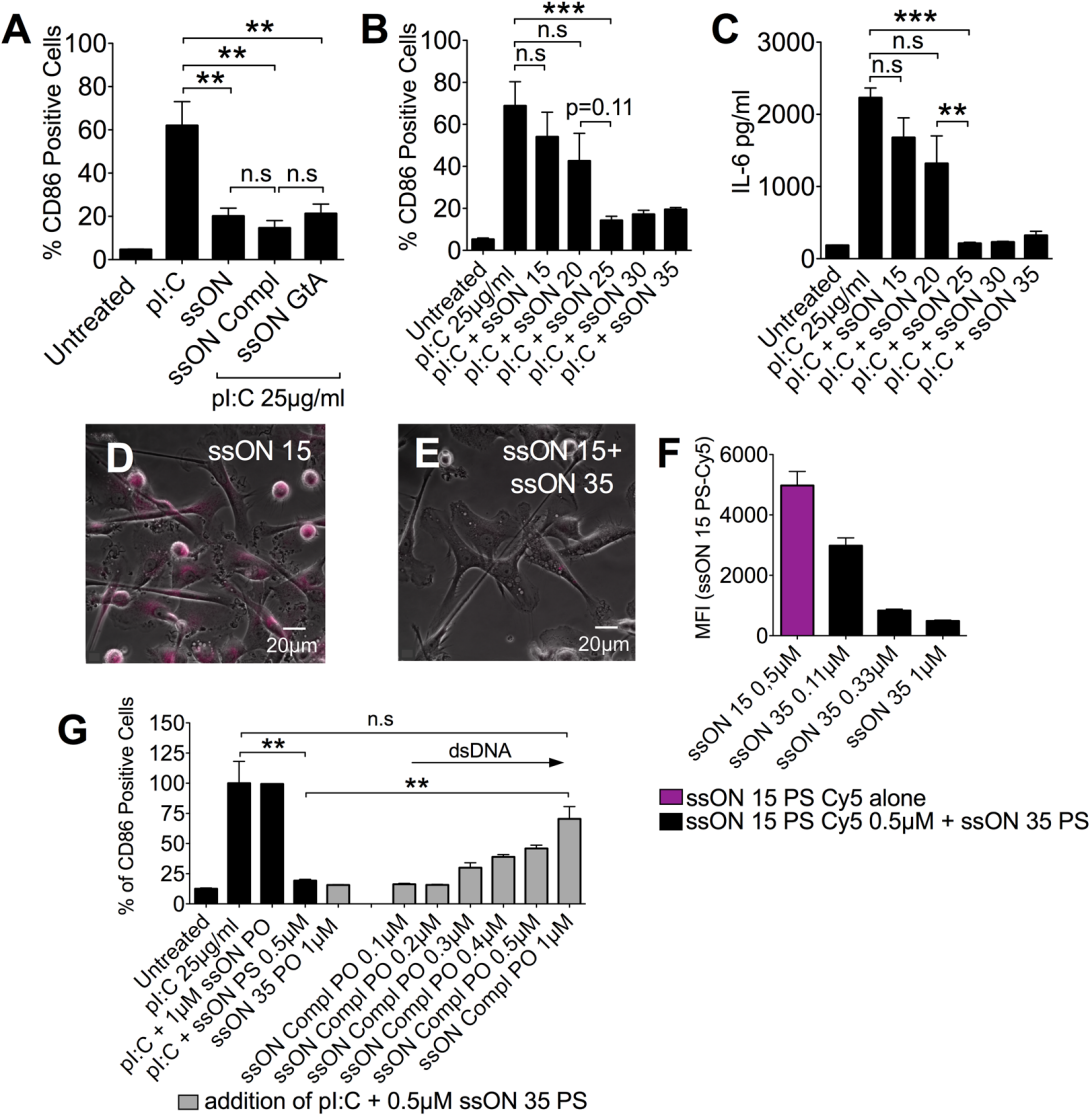
SsON, but not dsDNA, of at least 20–25 bases inhibit TLR3 activation. (**A**) The inhibition of pI:C-induced CD86 expression (48 h) in moDC was not dependent on a canonical sequence (for sequences, see Table S1). (**B**,**C**) The inhibition of pI:C-induced CD86 expression (48 h) (**B**) and IL-6 production (24 h) (**C**) in moDC was dependent on the ssON length (ssON PS 15–35). (**D**) Uptake of fluorescent ssON-color Cy5 15 PS in moDC (**E**) in the presence 0.5 µM ssON 35 PS. (**F**) Inhibition of fluorescent ssON 15 PS uptake was dependent on ssON 35 PS concentration. (**G**) Introduction of the complementary strand (ssON Compl PO) abolished the inhibitory effect on pI:C-induced CD86 expression (48 h) of 0.5 µM ssON 35 PS. All data are from at least three donors in duplicate. Error bars are given in SEM. Non-parametric Mann-Whitney test was used to compare the data. P-value: not significant (n.s) P > 0.05; *P ≤ 0.05; **P ≤ 0.01; ***P ≤ 0.001.

CpG motifs in DNA can bind to TLR9, leading to activation of TLR9^+^ cells such as plasmacytoid dendritic cells and B cells. We previously showed that a CpG containing ssON (35 bases) inhibited TLR3 activation in moDC lacking both TLR7 and TLR9^17^. In the present study, we have exclusively used ssON without CpG motifs to address their capacity to inhibit TLR3 activation. To get further insight as to whether the observed inhibition of endocytosis and downstream TLR3 activation could be linked to a particular ssON sequence, three different sequences were compared (Table S1). SsON GtA 35 PS was based on the parent sequence ssON 35 PS, but all the guanosine (G) bases were substituted for adenosine (A), while ssON Compl 35 PS is the complementary sequence to ssON 35 PS. All displayed the same inhibitory effect in moDC treated with pI:C for 48 h (Fig. 4A). To get further insights to the structural requirement, we assessed if the length of ssON influences its efficacy. We found that shorter ssON (Table S1) did not possess the same inhibitory capability as longer versions, with a length cut off between 20 and 25 bases for effective ssON (Fig. 4B,C). Therapeutic ssON for anti-sense purposes delivered without transfection agents (gymnotic delivery) are usually less than 20 bases^30,31^. Therefore, we next evaluated the uptake of ssON composed of 15 bases. Microscopy studies showed that fluorescently labelled ssON 15 PS was readily internalized by moDC and that this cellular uptake was efficiently blocked by the addition of ssON 35 PS (Fig. 4D,E) in a concentration dependent manner (Fig. 4F). Hence, these data provide clues as to why therapeutic anti-sense ssON that rely on endocytic uptake should be shorter than 20 bases.

We next investigated whether the inhibitory effect is induced by other classes of extracellular nucleic acids, or if it requires ssON. In order to assess if the introduction of double-stranded (ds)DNA influences endocytic uptake, ssON 35 PS was kept at a constant concentration (0.5 µM), while increasing concentrations of a non-stabilized complementary strand (ssON Compl 35 PO) were introduced (Fig. 4G). MoDC were treated with pI:C and the inhibitory activity of the ssON was reduced when the complementary strand was introduced. The inhibitory effect was absent when all ssON 35 PS had a complementary strand to hybridize to (ssON Compl PO 1 µM). Naturally occurring PO ssON is degraded unless it can hybridize to ssON 35 PS. A PO backbone containing ssON (ssON 35 PO) had no inhibitory effect on pI:C-induced maturation 48 h post initial treatment, consistent with its degradation. The inhibitory effect of ssON 35 PS remained with the addition of non-complementary ssON 35 PO (Fig. 4G). Altogether, these data show that ssON, but not dsDNA, can inhibit endocytic uptake and downstream effects of the TLR3 agonist pI:C in a concentration dependent manner, and that the effects are not linked to a specific ssON sequence or motif. However, the inhibition is dependent on the length of the ssON, with a full effect reached at or above 25 bases.

### Both ssDNA and ssRNA inhibit clathrin-dependent endocytic pathways

**Figure 5:**
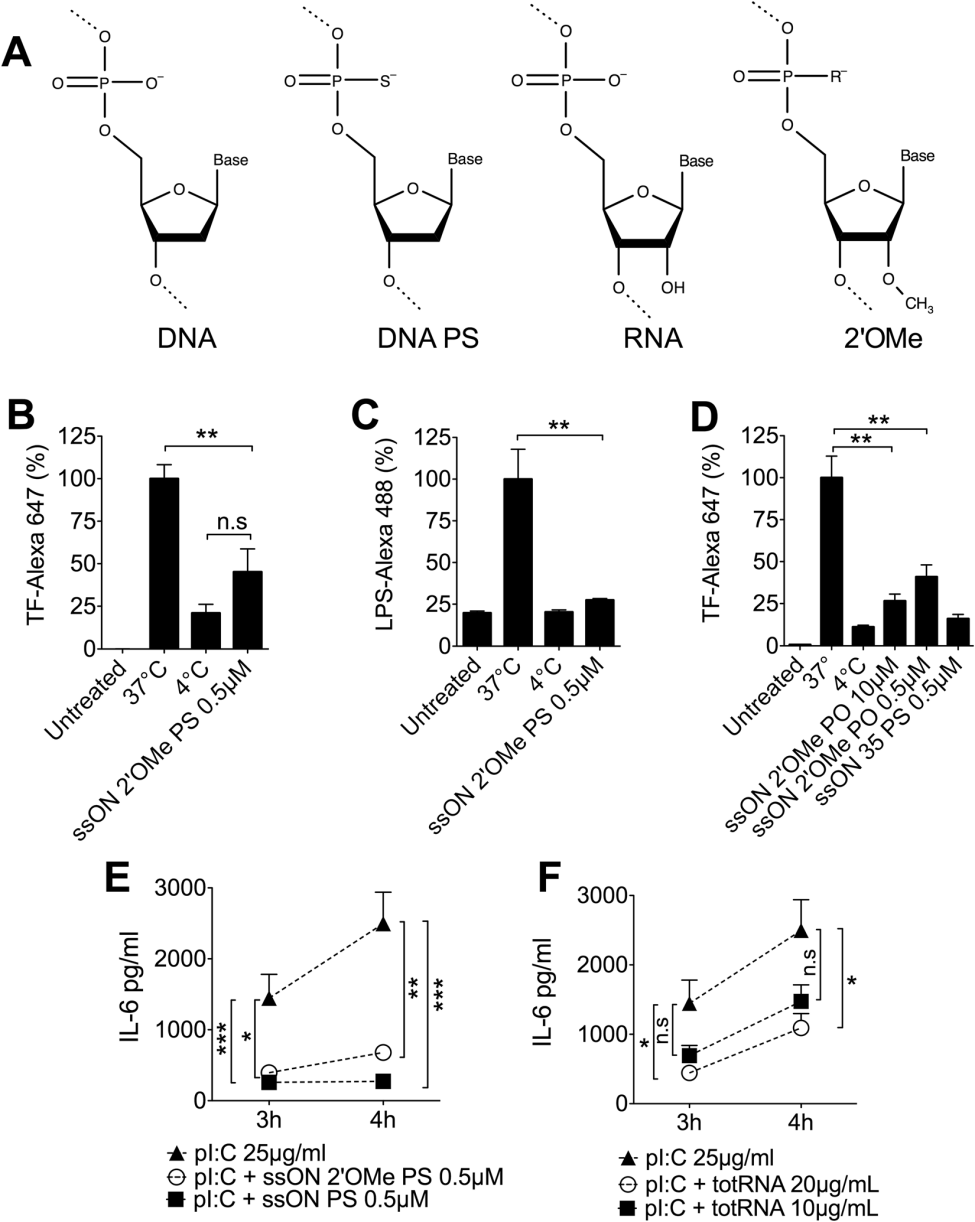
SsRNA display similar ability as ssDNA to inhibit endocytic uptake in human moDC. Human moDC were treated for 45 min at +37 °C or +4 °C with TF (Alexa 647) or LPS (Alexa 488) with or without addition of ssON 2′OMe RNA PS (Table S1). (**A**) Structures of repeating units of oligonucleotides and oligonucleotide analogues used in this study. (**B**,**C**) 0.5 µM ssON 2′OMe RNA PS inhibited uptake of fluorescently labelled TF and LPS. (**D**) SsON 2′OMe RNA with a native PO backbone is able to block TF uptake in moDC. (**E**) SsON 2′OMe RNA PS displayed the same efficacy as ssON 35 DNA PS in blocking pI:C-induced secretion of IL-6 from moDC. (**F**) Total RNA inhibited pI:C-induced IL-6 secretion from moDC in a concentration dependent manner. All data are from at least three donors in duplicate. Error bars are given in SEM. Non-parametric Mann-Whitney test was used to compare the data. P-value: not significant (n.s) P > 0.05; *P ≤ 0.05; **P ≤ 0.01; ***P ≤ 0.001.

To elucidate if the ability to inhibit endocytosis is exclusive for ssDNA, or if ssRNA also possess similar inhibitory capability, we synthesized a 35mer ON, comprised of the non-toxic, modification 2′-*O*-Methyl RNA (2′OMe) often found in ribosomal RNA and small nuclear RNAs (Fig. 5A) (Table S1). This ssON 2′OMe RNA analogue with a PS backbone effectively inhibited uptake of both TF and LPS (Fig. 5B,C). As with ssDNA, 2′OMe ssRNA with an unmodified PO backbone could inhibit TF uptake albeit with a lower efficacy (Fig. 5D). Further evidence of transient inhibition was found by showing decreased IL-6 secretion from moDC after incubation with pI:C and ssON 2′OMe PS, with a reduction comparable to ssON 35 PS (Fig. 5E). In addition, purified total RNA (totRNA), using a cut off filter below 200 bases, reduced production of IL-6 in a concentration dependent manner, when measured after 3–4 h (Fig. 5F). These data show that ssON, with the capacity to temporarily inhibit clathrin-mediated endocytic uptake in human moDC, can be of either ssDNA or ssRNA origin.

### No major changes in the transcriptome or proteome after inhibition of clathrin-mediated endocytosis in moDC

**Figure 6:**
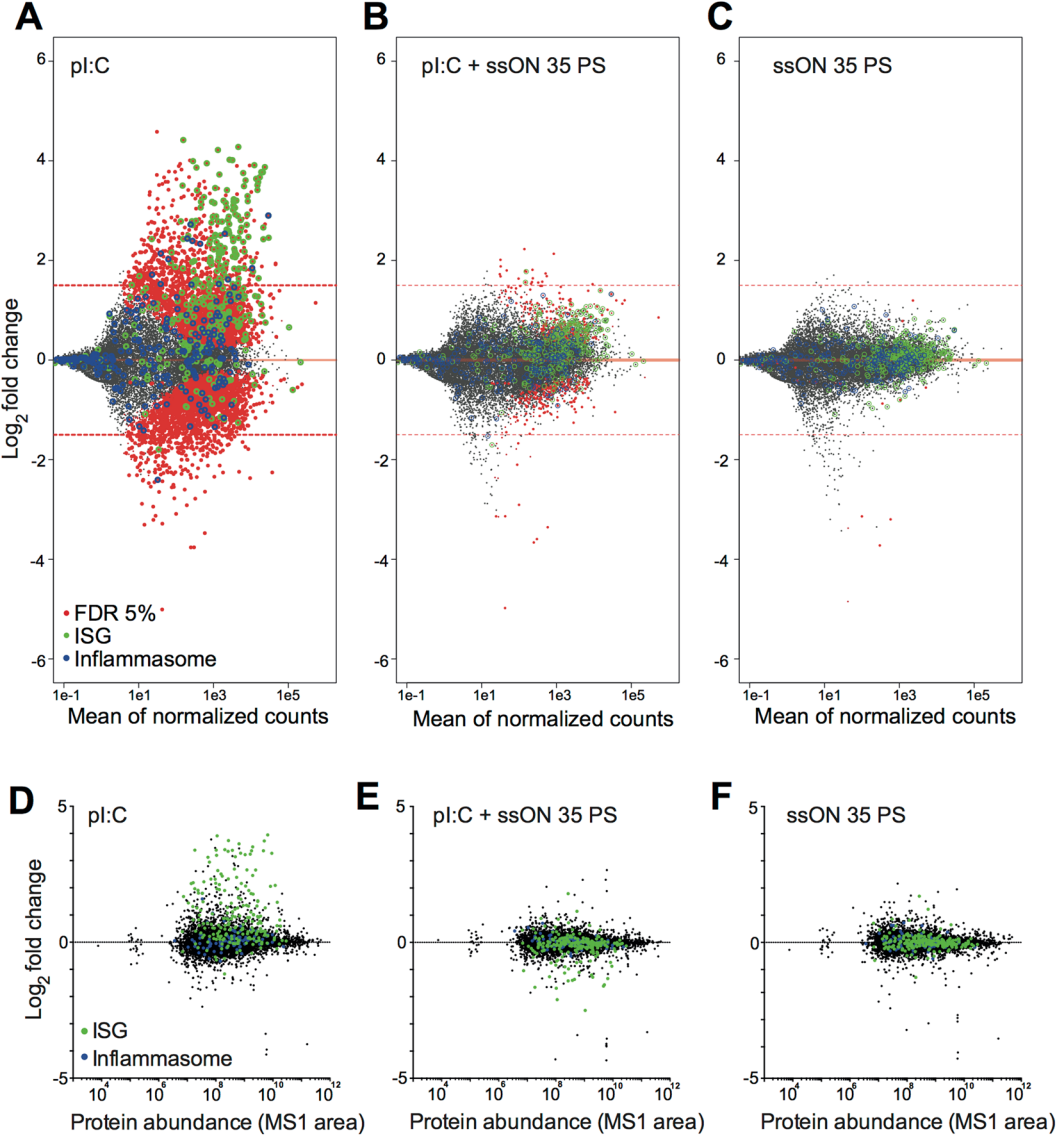
SsON 35 PS inhibits pI:C induced changes in the transcriptome and proteome profile in moDC. Colored circles mark genes included in specific categories (green = ISGs, blue = inflammasome related). (**A**–**C**) RNA-seq data from moDC 24 h post treatment (3 donors). MA-plots log2 fold changes adjusted p-values below 0.05 in red. (**A**) PI:C treatment vs non-treated cells shows high upregulation of ISGs. (**B**) Combined treatment with pI:C and ssON 35 PS (0.5 µM) vs non-treated cells show minor differences. (**C**) SsON 35 PS treatment (0.5 µM) vs non-treated cells show minor differences. (**D**–**F**) Whole cell proteomic data from moDC 24 h post treatment (3 donors). MA-plots of whole cell proteomic data (protein log intensity ratio versus average intensity). (**D**) PI:C treatment vs non-treated cells show high upregulation of ISGs. (**E**) Combined treatment with pI:C and ssON 35 PS (0.5 µM) vs non-treated cells show minor differences. (**F**) SsON 35 PS treatment (0.5 µM) vs non-treated cells show minor differences. See also Figs S3–S5.

To gain insight to the cellular response occurring after the shut-down of the clathrin-mediated endocytosis in moDC, we performed combined transcriptomic and proteomic studies after exposure to ssON (Figs 6 and S3). These studies were also conducted after stimulation with pI:C with and without ssON, to get an overall picture of ssON’s capacity to inhibit TLR3 activation (Figs 6 and S4). RNAseq analyses revealed that there was a strong upregulation of IFN-stimulated genes (ISG) but not inflammasome related genes^32^ in pI:C-stimulated moDC after 24 h (Fig. 6A). Ingenuity pathway analyses (IPA) showed upregulation of genes in the canonical pathways “Interferon signaling”, “Role of Pattern Recognition Receptors in Recognition of Bacteria and Viruses” and “Crosstalk between Dendritic Cells and Natural Killer Cells” (Fig. S5A related to Fig. 6A). The addition of ssON 35 PS abrogated the pI:C-mediated induction of ISGs, supporting an effective inhibition of TLR3 activation by ssON (Fig. 6B). Treatment with ssON 35 PS alone did not lead to any clear changes in the transcriptome, which was measured for up to 24 h (Figs 6C and S3A,B).

The proteomic analyses similarly showed up-regulation of ISGs, but not inflammasome-related proteins in pI:C-stimulated moDC after 24 h (Fig. 6D). Proteomic analyses confirmed that ssON 35 PS abrogated pI:C-mediated induction of ISGs (Fig. 6E). Stimulation with ssON 35 PS alone did not lead to any major changes of the proteome (Fig. 6F). The time course of overall proteome changes in moDC was measured at seven time-points; before stimulation, at 15, 30 and 60 min as well as 3, 8 and 24 h post treatment with ssON 35 PS (Fig. S3B,C). To select protein subsets with correlating protein expression levels for further interpretation of the data, hierarchical clustering and networks analyses were performed (Fig. S4A related to Fig. 6). IPA analysis revealed that pI:C stimulation of moDC resulted in a clear time-dependent up-regulation of protein subsets linked to “Interferon signaling”, “Role of Pattern Recognition Receptors in Recognition of Bacteria and Viruses” and “Crosstalk between Dendritic Cells and Natural Killer Cells” (Fig. S5B related to Fig. 6D), in accordance with the RNAseq data and previous studies^33,34^. Up-regulated proteins after pI:C stimulation included MHC class I and class II molecules, and markers associated with moDC maturation, such as CD83 (Fig. S4B). As expected, many molecules were detected that play a role after PRR engagements, interferon signaling and activation of IRF. Network analysis performed on the up-regulated cluster after pI:C stimulation identified IRF1, 7, 8 and STAT1 as the most interconnected proteins (>10 edges) (Fig. S4B), whereas the protein-level changes following ssON stimulation were less pronounced (Figs 6F and S3C). No clear trends or clusters could be detected in the protein subset of up- or down-regulated proteins after ssON stimulation of steady state human moDC. Altogether, these data show that stimulation of pI:C leads to up-regulation of ISGs detected at both the transcriptional and proteome level in moDC, while the addition of ssON blunted this response. Moreover, there were no major changes occurring in the transcriptome in moDC treated with ssON alone, with minor changes starting to occur in the proteome after 8–24 h. The lack of changes in the proteome and transcriptome does however not exclude that ssON induce other changes occurring in the cell. Modulations in the cytoskeleton, phosphorylation patterns or alterations in protein-protein interactions might all contribute to the SOMIE effect.

### Administration of ssON 35 PS modulates dsRNA-mediated inflammation in the skin of macaques

**Figure 7:**
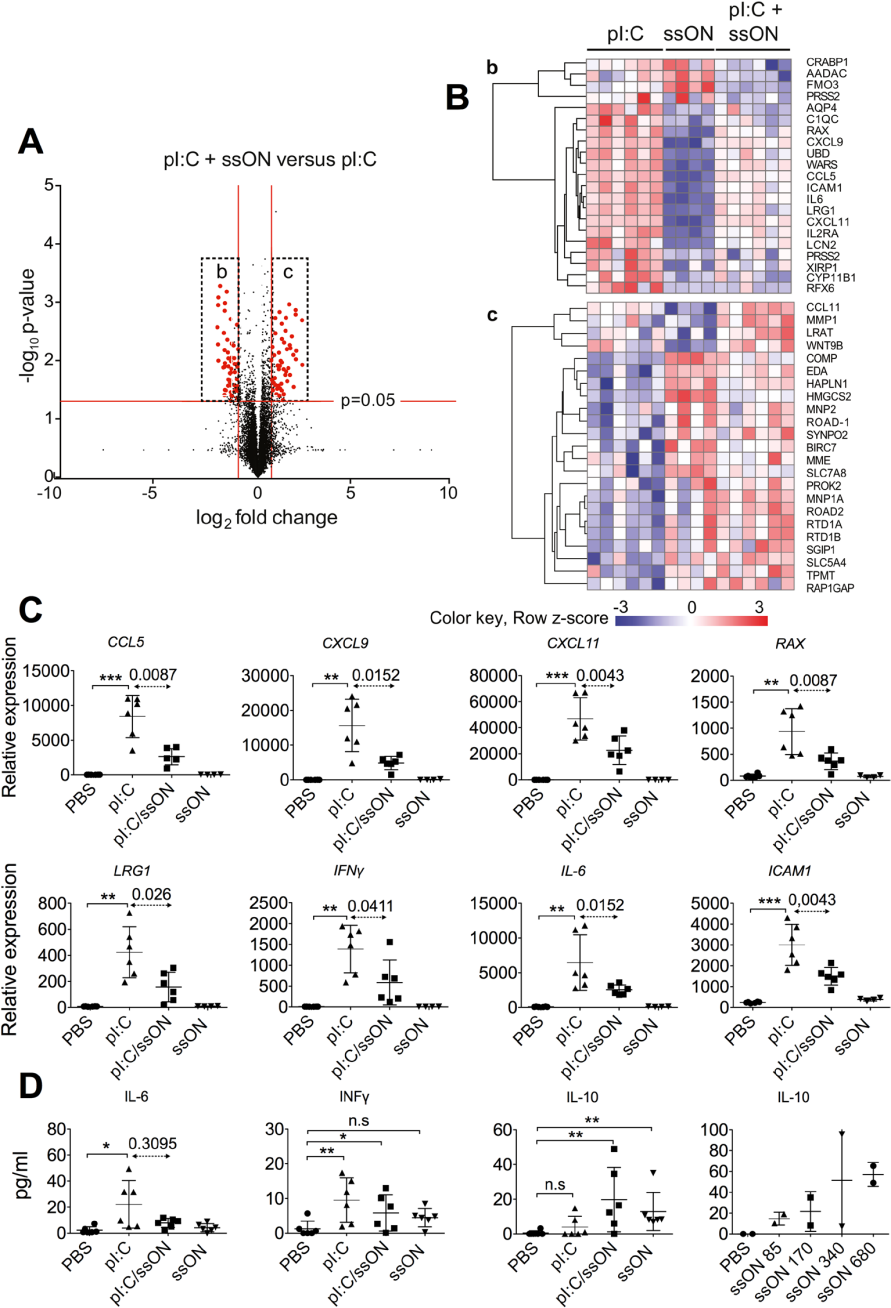
SsON dampens dsRNA-mediated inflammatory signatures in macaque skin. (**A**) RNA expression in skin following injection with dsRNA (pI:C) in the presence or absence of ssON 35 PS. Volcano plot illustrating log2 fold-change and p < 0.05 in red. (**B**) Clustering of genes showing the highest differential expression comparing pI:C + ssON with ssON 35 PS treatment alone (b,c). Gene expression is represented as gene-wise standardized expression (Z score) with p < 0.05. Red and blue correspond to up- and down-regulated genes, respectively. Unsupervised hierarchical clustering of genes based on Spearman-correlation. (**C**) Relative mRNA expression values from the microarray analyses of individual macaque skin biopsies 24 h post-stimulation (means ± SEM). (**D**) Concentrations of indicated cytokine proteins present in supernatants of enzymatically digested skin biopsies (24 h post-stimulation *in vivo*) (means ± SEM from individual animals). Lower right panel shows IL-10 production in a dose escalation experiment with ssON ranging from 85–680 μg per injection (n = 2). Significant differences were assessed by non-parametric Kruskal-Wallis test and Dunn’s post-test (*P < 0.05, **P < 0.01 and ***P < 0.001). Different treatment groups were compared using nonparametric Mann-Whitney unpaired test, as indicated. See also Figs S5 and S6.

To assess whether ssON 35 PS can mediate effects *in vivo*, we measured the local infiltration of cells and their innate immune signatures after intradermal injection of pI:C in the presence or absence of ssON 35 PS in macaque skin biopsies taken from the site of injection. Multicolor flow cytometry was used to phenotype cells isolated from epidermal and dermal layers^35,36^ (Fig. S6 related to Fig. 7) and revealed that injection of pI:C led to influx of polymorphonuclear neutrophils (PMN) and HLA-DR^+^ CD1^−^ antigen presenting cells (APC), while addition of ssON reduced the infiltration of epidermal HLA-DR^+^ CD1a^−^ cells, but not PMN (Fig. S7 related to Fig. 7).

To assess the global innate response to pI:C in the presence or absence of ssON, transcriptional profiling was performed on the skin biopsies. IPA analyses revealed that several pathways for innate immunity, such as “Communication between Innate and Adaptive Immune Cells”, “TREM1 signaling”, “Role of Pattern Recognition Receptors in Recognition of Bacteria and Viruses”, and “Crosstalk between Dendritic cells and Natural Killer cells”, were engaged after injection of pI:C (Fig. S5C related to Fig. 7). We next compared the group receiving intradermal injection of pI:C alone with the group receiving pI:C in combination with ssON, and calculated the fold change as well as significant differences between the two groups (Fig. 7A). The results show that among the top down-regulated genes, after addition of ssON 35 PS, are chemokines and genes implicated in inflammatory conditions (Fig. 7A). PI:C treatment alone resulted in significant induction of the chemokines *Ccl5, Cxcl9*, and *Cxcl1* as well as *Rax*, a cellular activator for dsRNA-dependent serine/threonine protein kinase^37^, which were all significantly reduced with combined pI:C and ssON treatment (Fig. 7B). Similarly, *Icam-1* and *Lrg1*, involved in cell adhesion, were upregulated in tissue after pI:C injection, but this induction was reduced by co-injection with ssON (Fig. 7B).

IFN-γ regulate many chemokines^38^. PI:C injection indeed resulted in increased expression of both *IFN-*γ and *IL-6*, while the combined pI:C and ssON treatment reduced this expression in concordance with modulation of chemokine expression (Fig. 7B). To validate cytokine protein secretion in the skin, aliquots of filtered-dermis supernatant were collected and, without additional *in vitro* stimulation, analyzed using bioplex analyses (Fig. 7C). Significant induction of IL-6 and IFN-γ was detected after pI:C treatment. There was a clear trend that addition of ssON decreased IL-6 and IFN-γ production and instead provoked significant IL-10 release. Notably, ssON alone induced dose-dependent IL-10 secretion *in vivo* (Fig. 7C). Altogether, these findings show that there was a local dampened inflammatory signature in animals that received combined pI:C and ssON treatment as compared with pI:C alone, in agreement with ssON’s ability to inhibit TLR3 activation.

## Discussion

Herein, we revealed that certain ssON can abolish uptake of ligands and thereby inhibit the activation of TLR3, 4 and 7 signaling endosomes, which supports and extends our previous publication that a CpG ssON (35 bases) inhibited TLR3 signaling^17^. We here also demonstrated that ssON block the uptake of TF, LDL, LPS and pI:C as shown by microscopy and quantified by flow cytometry. Functional inhibition of TLR3, 4 and 7 signaling was shown by measuring cytokine secretion and expression of co-stimulatory molecules from stimulated moDC and PBMC. We further assessed global changes by performing RNAseq and whole cell proteomic analyses, which support the block in endosomal signaling. Characterization of structural requirements for inhibition of clathrin-mediated endocytosis revealed that either ssDNA or ssRNA, but not dsDNA conferred the inhibition. The capability was not strictly dependent on the ssON sequence, but required that it was composed of at least 25 bases to have a full inhibitory effect. Hence, the inhibitory effect was not dependent on CpG motifs. We further provide evidence that ssON 35 PS modulated TLR3 activation *in vivo* in macaques by measuring local responses in the skin after injection.

The transient shut down of clathrin-mediated endocytosis did not severely hamper the cells. This was supported by the finding that cytokine secretion occurred in the presence of ssON provided that pI:C was taken up prior to the addition of ssON. Further, we show that ssON did not induce any apparent toxicity, and that it can rescue cells from pI:C-induced cell death. RNAseq and whole cell proteomic analyses supported this conclusion and no major changes occurred in the transcriptome or proteome after ssON treatment alone, which was measured for up to 24 h post stimulation. Injection of ssON in macaques did not induce inflammation, instead a dampening of dsRNA (pI:C)-mediated inflammation was observed. We also show that macropinocytosis (measured by uptake of dextran), was not affected by ssON, allowing continued uptake of nutrients in fluid phase.

Our findings that either ssDNA or ssRNA of at least 25 nucleotides have the capacity to temporarily (provided that they are not degraded) shut down clathrin-mediated endocytosis opens up intriguing questions in host-viral interactions, RNA biology and autoimmunity. Our data have implications for 1) viral endocytosis 2) cellular uptake of miRNA 3) role of stabilizing modifications of endogenous RNA 4) immune regulatory function of noncoding RNAs 5) development of ssON based therapeutics (anti-sense ONs, splice switching ONs, immunosuppressive ONs, siRNA, CpG adjuvant).

Altogether, these studies reveal a regulatory role for extracellular ssON in the endocytic uptake of TLR ligands and we have termed this regulatory process SOMIE (SsON-Mediated Interference of Endocytosis).

Infection by pathogens and cellular damages lead to an accumulation of extracellular nucleic acids that can be degraded by RNases and DNases, but also internalized into endosomes for subsequent TLR activation. To avoid excessive endosomal TLR activation under circumstances of heavy load on these systems, it is crucial to tightly control innate immune responses, thereby mitigating potential self-destructive events. An increasing number of molecules such as A20, CYLD, USP4, USP25, OTUB1/2, DUBA, and MYSM1 have been implicated in the dampening of intracellular signaling cascades triggered by PRR^39^. However, there is a growing appreciation that the effects exerted by these enzymes cannot solely explain the rigorous regulation of PRR activation. Here we demonstrate that ssON, both native ssDNA and ssRNA 2′OMe, temporarily interfere with CME and downstream endosomal TLR activation. SOMIE serves as a regulatory process to temporarily limit activation of the innate immune system. Treatment with stabilized ssON 35 PS displayed a more prolonged block of endocytosis, while the MPC remained unaffected, showing that the inhibitory effect was specific for certain endocytic pathways. Phagocytosis is known to be controlled by both activating (prophagocytic; ‘eat me’) and inhibitory (‘don’t eat me’) receptors. The inhibitory signal-regulatory protein (SIRP)α provides a blockade of phagocytosis after binding to the ligand CD47, a checkpoint of phagocytosis^40^.

Endocytosis of cargo was quantified by flow cytometry and visualized by microscopy, while functional activation of TLRs were measured by induction of co-stimulatory molecules CD80 and CD86 in moDC and secretion of cytokines. To reveal if ssON specifically affect activation from TLRs localized in endosomal compartments, we selected TLR agonists with distinct properties and assessed if ssON 35 PS inhibited secretion of IL-6. The TLR7/8 agonist R848 is membrane permeable^5^ and was not affected by the addition of ssON. In comparison, the activity of the endocytosis-dependent TLR7 agonist CL307 was significantly inhibited by ssON 35 PS. Furthermore, we studied LPS-sensing TLR4 that has two distinct signaling pathways in moDC. SsON 35 PS selectively decreased LPS-mediated secretion of IL-29/CXCL10, which is primarily downstream of TRIF and dependent on endocytosis, while it left the MyD88-dependent IL-6/IL-10 secretion unaffected. It remains to be further elucidated in which cell types TLR4 are coupled to both the Myd88 and TRIF signaling pathways and to reveal potential species differences.

Kinetic analyses revealed that the inhibitory capability of ssON 35 PS decreased if added more than 50 min post stimulation with pI:C and was almost negligible after 2 h. It therefore seems likely that ssON cannot inhibit ongoing TLR3 signaling. When treating moDC in the reverse fashion, starting with a single treatment of ssON 35 PS, pI:C-induced maturation was completely blocked for up to 24 h. PS substituted ssON have a half-life in serum-containing cell culture media that exceed 24 h^19^. PI:C, on the other hand, is rapidly degraded in serum containing media^41^ and was not able to trigger TLR3 in moDC after pre-treatment with ssON 35 PS. It can be envisioned that the interference with endocytic activity allows moDC to respond to cargo recently taken up, without receiving excessive additional stimulus or contra-orders prior to fulfilling the response to the first endocytosed cargo. In addition, the finding that moDC can mature and produce IL-6 production in the presence of ssON, provided that the cargo (pI:C) was first allowed to be taken up, suggests that a temporal shut-down of clathrin-mediated endocytosis does not severely impair the cell. Transcriptomic and proteomic moDC data provided further evidence that ssON effectively blunted the pI:C-mediated response and that no major changes occurred in ssON treated cells for up to 24h.

We synthesized a panel of ON (Table S1) to determine the structural requirements for SOMIE. The SOMIE effect was not strictly dependent on the sequence or motif because the complementary strand to ssON 35 PS and a mutated ssON, in which all G were changed to A, showed similar efficacy. The interference with endosomal TLR activation by ssON was potent with an IC_50_ of about 150 nM. This led us to speculate that we are monitoring a biologically conserved function. However, to our knowledge the source of ssDNA in the cell is limited and primarily found in exosomes^42^, while ssRNA on the other hand are abundant. We demonstrated that a 35mer fully PS substituted RNA analogue 2′OMe ssON has the ability to block pI:C-induced moDC maturation and that total RNA (totRNA) inhibited pI:C-induced secretion of IL-6. Taken together, the SOMIE activity is not strictly dependent on ssON sequence, can occur with both ssDNA and ssRNA, and stability modifications (i.e. PS) are not essential for inhibition, but needed for retained activity over time. It remains to be further elucidated whether the metabolism of oligonucleotides and differential addition of stabilizing base modifications, such as 2′OMe or locked nucleic acid (LNA), can influence susceptibility to autoimmunity by setting the threshold of endosomal TLR activation.

The mammalian cell contains a plethora of both coding and non-coding ONs with varying lengths^43^. Immuno-regulatory micro RNAs (miRNA) consist of 18–23 bases ssON^44^. However, cells also contain ssON that are longer in size^43^. During cell death with release of cellular content, it is conceivable that both coding and non-coding DNA and RNA of varying length can be released into the extracellular space, becoming accessible to degradation by DNases and RNases. It can be postulated that the activity of these enzymes will greatly affect the size composition of ONs in the extracellular space, and ultimately affect triggering of nucleic acid sensing PRR. We here show that there is a length requirement of at least 20–25 bases to exert SOMIE activity. SsON (25–35 bases) inhibited uptake of shorter ssON (15 bases), while ssON 15 PS did not display SOMIE activity. This is consistent with recent data suggesting that miRNA (18–23 bases) released into the circulation and extracellular space during ischemia may instead trigger cytokine production via endosomal TLR7/MyD88 signaling^45^. The revealed length requirement for SOMIE allude to an unknown function of longer non-coding RNAs and shows that miRNA are too short to provide endocytic checkpoint control.

Our finding that ssON of at least 20–25 bases interfere with endocytosis provides a rational as to why ssON used as antisense, or exon skipping ssON with gymnotic delivery, should be shorter than 20 bases to allow cellular uptake through endocytic pathways. Similarly, CpG ssON aimed for TLR9 agonistic actions should be less than 20 bases^46^, to avoid inhibition of endocytosis, which may inadvertently cause off-target effects in cells lacking TLR9^17^. It is conceivable that the size and the relative ds:ss composition of ON in the extracellular space will depend on the local microbial milieu, with an overflow of ON during infections. To our knowledge, there is currently no technique available to enable measurement of the intracellular or extracellular concentrations of naturally occurring ON with varying length. Hence, to what extent and the timeframes for the observed ssON-mediated TLR inhibition occurring by natural ligands cannot be truly estimated at the present time. Nevertheless, we provide evidence that ssON 35 PS exerted immunomodulatory effects *in vivo* after intradermal injection in macaques. Skin biopsies showed dampened pI:C-mediated inflammation in the dermis of macaques after injection of ssON and reduced secretion of IL-6 was confirmed in supernatants obtained from skin biopsies *ex vivo* (without additional *in vitro* stimulation). Notably, ssON induced dose-dependent IL-10 secretion *in vivo*, suggesting that inoculation with ssON may shift the direction of local immune responses. It remains to be elucidate which cell type(s) in the skin produce IL-10 after ssON treatment. We did not find any signs of IL-10 production in moDC in the genomic and proteomic studies after stimulation with ssON. We hypothesize that the local environment and cellular cross-talk might be pivotal for induction of IL-10 and a wide range of cell populations can be induced to produce human IL-10, including T cells (Tregs, Th1, Th2, and Th17), subsets of DC, macrophages, neutrophils, B cells, mast cells, fibroblasts, and keratinocytes^47^.

Immunosuppressive ssON have been suggested to dampen or prevent diseases characterized by pathologic immune stimulation and autoimmunity^48–50^. Several immunosuppressive ssON were previously shown to contain sequences or motifs required for direct binding, to for example, TLR7 or TLR9^14,51^, or repeats (TTAGGG) that interact with STAT1 and STAT4^52^. We, and others, have previously suggested that TLR3 signaling can be inhibited by ssON in order to dampen over-reactive responses contributing to TLR3 linked pathogenesis^17,18^. Here we provide evidence that ssON might have a broader immune regulatory role than previously anticipated by acting as gatekeepers for uptake of cargo into endosomes, thereby providing a fluctuating threshold for potential autoimmune reactions triggered by endosomal TLR3/4/7 activation.

## Material and Methods

### Primary human cells

Human Buffy coats were acquired from Karolinska Institutet, Stockholm, Sweden. All experimental work with human peripheral blood cells were carried out in accordance with Swedish guidelines and regulations. All experimental protocols were approved by the local ethical committee in Stockholm (“Regionala etikprövningsnämnden i Stockholm”, Ethical permit Dnr 2006/229-31/3). According to regulations in Sweden, experimental *in vitro* work with cells from buffy coats does not require informed consent. Human monocytes were generated using the RosetteSep Monocyte Enrichment Kit (1 mL/10 mL buffy coat; StemCell Technologies) and differentiated into moDC, with GM-CSF (250 ng/mL; PeproTech) and IL-4 (6.5 ng/mL; R&D Systems) for 6 days in +37 °C, 5% CO_2_ at a density of 5 × 10^5^ cells/mL in RPMI 1640 completed with 10% FCS, 1 mM sodium pyruvate, 10 mM HEPES, 2mM L-glutamine, and 1% streptomycin and penicillin (all from Invitrogen Life Technologies) as previously described^17^. Differentiation of the cells was monitored by CD1a expression. PBMCs were isolated from buffy coats after Ficoll separation (Stemcell).

### Oligonucleotides

Fully PS-substituted ssON 35 PS and 2′OMe PS/PO were made by Integrated DNA Technologies. Other modified oligonucleotides were purchased from Eurofins. For sequences, see S Table 1. Total RNA was purified from moDC using RNeasy total RNA purification kit, according to manufacturer’s instructions (Qiagen).

### TLR ligands and labelled endosomal markers

TLR3 ligand pI:C (25 µg/mL unless otherwise stated), TLR7 ligand CL307 (1 µg/mL) and TLR7/8 ligand R848 (1 µg/mL) were all purchased from Invivogen. LPS (100 ng/ml) was purchased from Sigma. Transferrin-Alexa647, Dextran-Texas red 10 kDa, and LPS-Alexa488 were all purchased from Molecular probes. Cy3 labelled pI:C was generated using an oligonucleotide Label IT-kit (Mirus), according to manufacturer’s instructions.

### Uptake studies in moDCs

MoDC were exposed to endosomal markers, with or without addition of ssON, on ice in complete 10% RPMI media (or serum free media for PO ON uptake studies), and then transferred to +37 °C for 45 min. Cells were washed with cold PBS and fixed (Cytofix, BD Bioscience). Fluorescent signal was monitored by flow cytometry (Fortessa, BD Biosciences). Data were analyzed with FlowJo software (Tree Star, version 9.6.4). CME was monitored by: 25 µg/ml Transferrin-Alexa647, 5 µg/ml LDL-Dil or 1 to 5 µg/ml pI:C-Cy3. MPC: 0.5 mg/ml 10 kDa Dextran-Texas-Red. Endocytic uptake of TLR4: 1 µg LPS-Alexa488. Transfection of pI:C-Cy3 was performed with LyoVec (Invivogen) in a ratio 1:6 according to manufacturer’s instructions. For microscopy, moDC were adhered poly-L-lysine coated glass slides for 2–4 h. Cells were treated with ligands at +37 °C for 45 min in the presence or absence of 0.5 µM ssON 35 PS. Cells were washed and acquired at +37 °C in a wide-field Cell Observer microscope (Zeiss) using the 40X lens. Images were acquired and analyzed using the SlideBook 6 program (Intelligent imaging innovations). Alternatively, cells were treated with Poly I:C-Cy3 and Poly I:C-Cy3 with LyoVec the presence or absence of 0.5 µM ssON 35 PS at +37 °C for 40 min, after cell adherence. Cells were washed with PBS and stained with Wheat Germ agglutinin Alexa633 (Invitrogen) for 10 min. Cells were washed prior to fixation in 3.7% Formaldehyde (Sigma). Images were acquired in a LSM800 airy scan confocal microscope (Zeiss) using the 63X oil lens and the images were analyzed using the Zen blue software (Zeiss).

### MoDC maturation studies

MoDC were exposed to pI:C (25 µg/ml), CL307 (1 µg/ml), R848 (1 µg/ml) LPS (100 ng/ml), or pI:C/LyoVec (1 µg/ml) (Invivogen) with or without the addition of ssON, and assessed 48 h later by flow cytometry using monoclonal antibodies (Abs) targeting CD1a and the moDC maturation markers CD86, CD83, and CD80 (CD1a-Bv510, CD86-APC, CD83-FITC, CD80-PE; all from BD Biosciences). Where chloroquine was used, cells were pre-incubated with 10 µM chloroquine for 1 h at +37 °C. Dead cells were excluded using Live/Dead fixable near-IR dead cell stain kit (Life Technologies).

### Cytokine/Chemokine secretion studies

Supernatants were collected at given time points post treatment and secretion of cytokines/chemokines was measured by standard ELISA according to manufacturer’s instructions (Mabtech; IL-6, IL-10, and IL-29. Life Technologies: CXCL10) by a SpectraMax i3x, Molecular devices.

### TLR3 activation studies in HEK cells

TLR3 transfected HEK-blue™ cells (Invivogen) were seeded at 20 000 cells/well in 384 well plate (Corning) and left to attach for 1 h. Cells were treated with 1 µg/ml pI:C, specified concentrations of ssON 35 PS (Fig. S1) and incubated over night. TLR3 activity was measured at 640 nm in HEK-blue™ detection medium (Invivogen) with the Envision Plate Reader (Perklin Elmer), and given as % of pI:C treated cells without ssON 35 PS.

### Nuclease degradation of ssON 35 PO and PS

Nuclease degradation was performed similarly to Ciafre *et al*.^19^, and carried out in serum free RPMI media. DNase I (Qiagen) was used at a ratio of 1U/µg DNA. The reaction was carried out at +37 °C for 0–24 h at a concentration of 10 µM ssON 35 PO/PS. Aliquots were removed at given time-points, frozen at −80 °C and later analyzed on a 2% agarose gel. Electrophoresis was performed in TBE buffer for 1 h at 100 V. The gel was stained with ethidium bromide (0.5 μg/ml) and photographed using a gel doc ez imager (Biorad).

### Kinetic analysis of TLR3 inhibition

MoDC were exposed to 25 µg/ml pI:C/0.5 µM ssON 35 PS and then pulsed with either 0.5 µM ssON 35 PS or 25 µg/ml pI:C respectively over a time period from 5 min to 24 h. Cells were analyzed by flow cytometry 48 h post initial exposure for expression of maturation markers CD80 and CD86 in CD1a positive cells. Additionally, supernatants were collected 48 h post treatment and IL-6 was measured by ELISA (Mabtech).

### Viability test

Viable cells were assessed using a Live/Dead fixable near-IR dead cell stain kit 48 h post initial treatment with 25 µg/ml pI:C/0.5 µM ssON 35 PS according to manufacturer’s protocol (Life Technologies). Data was acquired by Flow cytometry. WST-1 assay was preformed according to the manufacturer’s protocol (Sigma) and measured on SpectraMax i3x.

### MoDC maturation studies with dsON

SsON 35 PS was pre-incubated at 5 µM with complementary ssON 35 PO at 1–10 µM in PBS at 55 °C and then left at ambient temperature for 30 min. ON mixture was added to moDC in a final concentration of 0.5 µM ssON 35 PS in combination with 25 µg/ml pI:C. MoDC maturation was assessed as described above.

### RNA sequencing and differential expression analysis

Total RNA was purified from moDC using RNeasy total RNA purification kit, according to manufacturer’s instructions (Qiagen) and submitted to the National Genomics Infrastructure Sweden Stockholm (NGI) for sequencing. Bioanalyzer traces and concentration values were obtained following the guidelines in the *Sample requirements for genomics* documentation available at the NGI website. The RNA sequencing was performed with the TruSeq RiboZero kit from Illumina. For detailed protocol, see supplementary Material and Methods section.

### Mass spectrometry based proteomics analysis of moDC

2 × 8 samples were collected at given timepoints, after moDC stimulation with either pI:C (25 µg/ml) or ssON 35 PS (0.5 µM), and snap frozen. For detailed protocol of sample preparations and protocol, see supplementary Material and Methods section.

For a complete Liquid chromatography tandem mass spectrometry (LC-MS/MS) analysis was performed using an Agilent 1200 nano-LC system coupled online to a Q Exactive Orbitrap (Thermo Fischer Scientific). The software Proteome Discoverer vs. 1.4.0.288 including Sequest-Percolator for improved identification^53^ was used to search the data against the human Ensembl database (version 37) for protein identification (false discovery rate (FDR) of <1%).

### Hierarchical clustering and network generation

The analyses were performed using the software Morpheus (https://software.broadinstitute.org/morpheus/index.html) on log_2_ transformed data. Network analysis to identify directly interconnected genes within the hierarchical clusters was performed using MetaCore™ version 6.22 (Thomson Reuters), and results displayed using Cytoskape version 3.2.1^54^.

### Animals and injections

Adult cynomolgus macaques (*Macaca fascicularis*) (n = 18, both females and males), were handled in accordance with European guidelines for NHP care (EU Directive N 63/2010). This study was approved and accredited under statement number 12-013 by the Ethical Animal Committee of the CEA “Comité d’Ethique en Expérimentation Animale” registered by the French Research Ministry under number 44. Animals were handled under sedation and intradermal injections were done in the upper left and right back flank with 170 µg of pI:C alone or with 170 µg of ssON 35 PS in 100 µL of PBS, or PBS alone. Alternatively, a dose escalation with ssON 35 PS was performed as indicated in figure legends. Skin biopsies (8 mm in diameter) were collected from anesthetized animals 24 h after injection.

### NHP tissue collection and flow cytometry

Cells were extracted from fresh skin biopsies collected 24 h after injections. For detailed sample preparation and protocol, see supplementary Material and Methods section.

Epidermal and dermal cells were stained with a mix of monoclonal antibodies (HLA-DR-V500, CD123-PECy7, CD45-V450, CD11c-APC, CD14-APC-H7 from BD Bioscience; CD66-APC, CD66-FITC from Miltenyi; CD1a from DAKO; CD163-PcPCy5.5 from Biolegend) and acquired by flow cytometry.

### Histochemistry

Live CD66^+^ CD45^+^ NHP cells were FACS-sorted (FACSAria, BD Biosciences) and put on Superfrost^®^ Plus gold slides (Thermo Scientific) by cytospin and stained using Giemsa according to clinical routine procedures at the Clinical Pathology Laboratory at Karolinska University Hospital Huddinge.

### Cytokine secretion assays

Aliquots of filtered-dermis supernatants isolated *ex vivo*, without further stimulation, were measured with the MILLIPLEX MAP NHP Cytokine Magnetic Bead Panel (Millipore, France) on a Bio-Plex device (Bio-Rad, France).

### Microarray analysis

Whole skin RNA was extracted from macaque skin biopsies. For detailed sample preparation and protocol, see supplementary Material and Methods section. RNA was hybridized to Agilent Rhesus Macaque Gene Expression Microarrays v2 for 17 h at 65 °C in a rotating Agilent hybridization oven. Microarrays were washed and scanned on the Agilent DNA Microarray Scanner (G2505C) using one color scan setting for 4 × 44 K array slides (Scan Area 61 × 21.6 mm, Scan resolution 5 µm, Dye channel is set to Green, PMT is set to 100%). Images were analyzed with Feature Extraction Software 10.7.3.1 (Agilent) using default parameters to obtain a background adjusted signal (gProcessedSignal) for each gene. The signals were subsequently quantile normalized to increase inter-sample comparability.

### Pathway analysis

Ingenuity Pathway Analysis software (Build version 456367 M, Content version 39480507 release date 20170914) (Ingenuity Systems) was used to identify canonical signaling pathways. To calculate significance of enrichment (Fisher´s exact test, performed within the software), the reference molecule set was Ingenuity Knowledge Base (Genes only). Three input molecule sets depending on analysis; (i) proteome clusters of proteins with correlating expression (mass spectrometry data, human moDC *in vitro*) selected based on hierarchical clustering (ii) RNAseq data (human moDC *in vitro*) filtered by absolute log_2_ fold change above 2 and (iii) microarray data from macaque skin biopsies filtered by absolute log_2_ fold change above 5. Gene Ontology (GO) enrichment analyses were performed using the GOrilla (Gene Ontology enRIchment anaLysis and visuaLizAtion tool) web tool^55^.

### Quantification and statistical analysis

Non-parametric Kruskal-Wallis unpaired test followed by Dunn’s post-test or Mann-Whitney test was used to compare the presented data in Figs 1–5 and 7B. Data for statistical calculations are from at least three donors in biological replicates in independent experiments. Data from macaque studies are shown for individual animals. Statistical calculations for transcriptomic and proteomic data are described above. P-value: not significant (n.s) *P* > 0.05; *P ≤ 0.05; **P ≤ 0.01; ***P ≤ 0.001. Statistics were calculated using GraphPad Prism 7 software.

## Electronic supplementary material

Supplementary information
