# Supplementary material for: Single-Stranded Nucleic Acids Regulate TLR3/4/7 Activation through Interference with Clathrin-Mediated Endocytosis
Source: Sci Rep. 2018 Oct 26;8:15841. doi: 10.1038/s41598-018-33960-4 (PMC6203749; doi:10.1038/s41598-018-33960-4)
Supplement: Supplementary file 1 — Supplementary information [file 41598_2018_33960_MOESM1_ESM.docx]

**Supplementary information to:**

**Single-Stranded Nucleic Acids Regulate TLR3/4/7 Activation through Interference with Clathrin-Mediated Endocytosis**

Peter Järver^a,1^, Aleksandra Dondalska^a^, Candice Poux^a^, AnnSofi Sandberg^b^, Joseph Bergenstråhle^c^, Annette E. Sköld^a^, Nathalie Dereuddre-Bosquet^d^, Fréderic Martinon^d^, Sandra Pålsson^a^ , Eman Zaghloul^e^, David Brodin^f^, Birgitta Sander^g^, Kim A. Lennox^h^, Mark A. Behlke^h^, Samir EL-Andaloussi^e,i^, Janne Lehtiö^b^, Joakim Lundeberg^c^, Roger LeGrand^d^, Anna-Lena Spetz^a,1,2^

^a^Department of Molecular Biosciences, Wenner-Gren Institute, Stockholm University, 106 91 Stockholm, Sweden

^b^Cancer Proteomics Mass Spectrometry, Department of Oncology-Pathology, Science for Life Laboratory, Karolinska Institutet, 171 65 Stockholm, Sweden

^c^Department of Gene Technology, Science for Life Laboratory, Royal Institute of Technology, 171 65 Solna, Sweden

^d^CEA, -Université Paris Sud-Inserm U1184, IDMIT Department, Institut de Biologie Francois Jacob (IBFJ), 922 60 Fontenay-aux-Roses, France

^e^Clinical Research Center, Department of Laboratory Medicine, Karolinska Institutet, 141 86 Stockholm, Sweden

^f^Bioinformatics and Expression Analysis core facility, Department of Biosciences and Nutrition, Karolinska Institutet, 141 83 Stockholm, Sweden

^g^Division of Pathology, Department of Laboratory Medicine, Karolinska Institutet, 141 86 Stockholm, Sweden

^h^Integrated DNA Technologies Inc, Coralville, 52241 Iowa, USA

^i^Department of Physiology, Anatomy and Genetics, University of Oxford, OX1 3PT Oxford U.K

^1^Correspondence: peter.jarver@su.se and anna-lena.spetz@su.se,

^2^Lead contact

**Supplementary Table 1:**

| **S Table 1** |  |  |
| --- | --- | --- |
| **Name** | **Sequence** | **Length** |
|  |  |  |
| ssON 35 PO | GAAGTTTTGAGGTTTTGAAGTTGTTGGTGGTGGTG | 35 |
| ssON 35 PS | G*A*A*G*T*T*T*T*G*A*G*G*T*T*T*T*G*A*A*G*T*T*G*T*T*G*G*T*G*G*T*G*G*T*G | 35 |
| ssON 30 PS | A*G*T*T*T*T*G*A*G*G*T*T*T*T*G*A*A*G*T*T*G*T*T*G*G*T*G*G*T*G | 30 |
| ssON 25 PS | T*T*T*G*A*G*G*T*T*T*T*G*A*A*G*T*T*G*T*T*G*G*T*G*G | 25 |
| ssON 20 PS | T*G*A*G*G*T*T*T*T*G*A*A*G*T*T*G*T*T*G*G | 20 |
| ssON 15 PS | G*G*T*T*T*T*G*A*A*G*T*T*G*T*T | 15 |
| ssON GtA PS | A*A*A*A*T*T*T*T*A*A*A*A*T*T*T*T*A*A*A*A*T*T*A*T*T*A*A*T*A*A*T*A*A*T*A | 35 |
| ssON Compl PO | CACCACCACCAACAACTTCAAAACCTCAAAACTTC | 35 |
| ssON Compl PS | C*A*C*C*A*C*C*A*C*C*A*A*C*A*A*C*T*T*C*A*A*A*A*C*C*T*C*A*A*A*A*C*T*T*C | 35 |
| ssON 2’OMe PO | *GAAGUUUUGAGGUUUUGAAGUUGUUGGUGGUGGUG* | 35 |
| ssON 2’OMe PS | *G*A*A*G*U*U*U*U*G*A*G*G*U*U*U*U*G*A*A*G*U*U*G*U*U*G*G*U*G*G*U*G*G*U*G* | 35 |
|  |  |  |
|  | All sequences written 5’ to 3’ * = PS *italic* = RNA _ = 2’OMe |  |


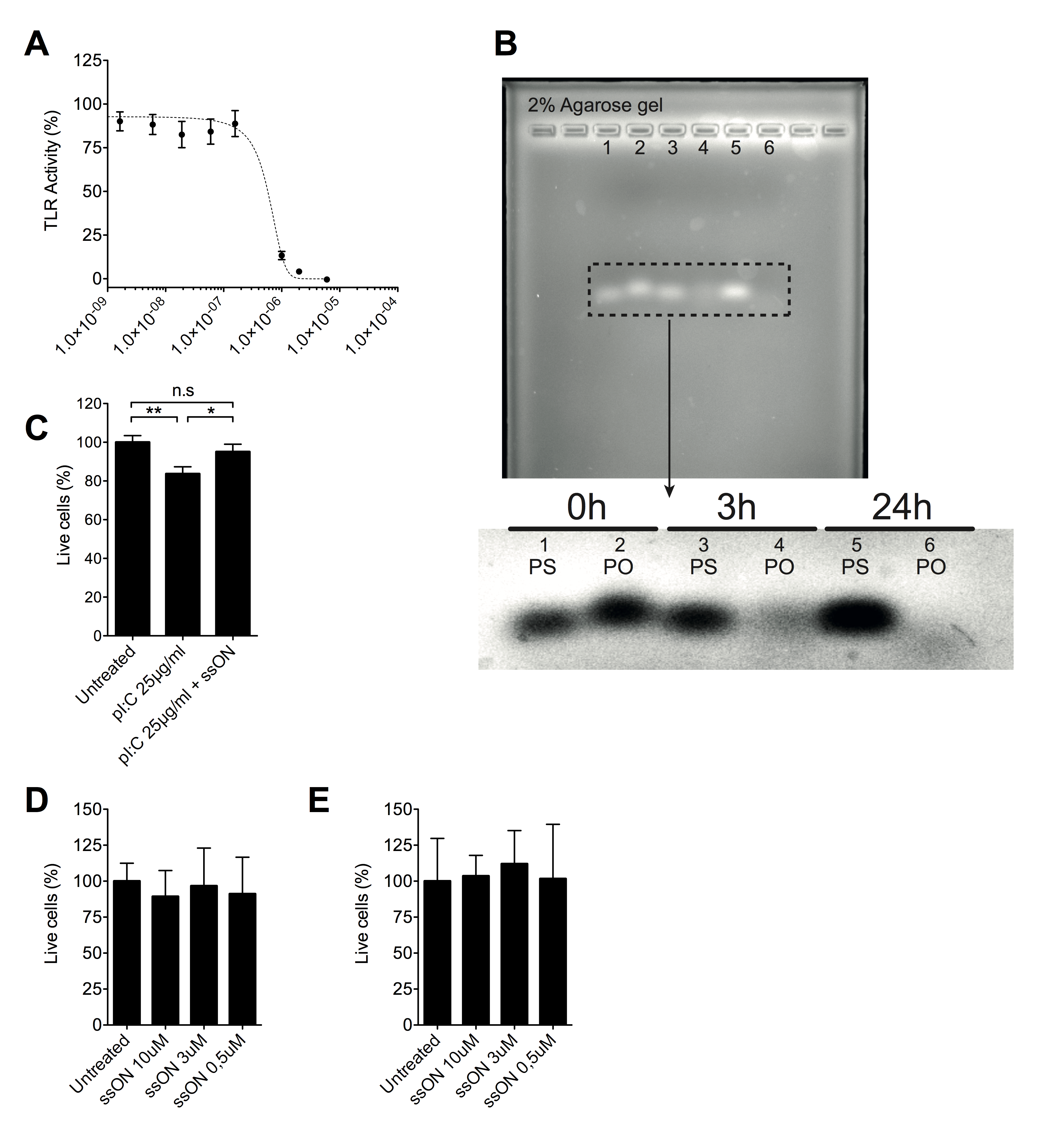


**S Fig 1. Related to Fig 2.** HEK cells, oligonucleotide stability and viability test

*(A)* Dose-dependent inhibition of TLR3 activation in TLR3 transfected HEK-Blue cells by ssON (24h). Cells treated with 1μg/ml pI:C and indicated concentrations of ssON35 PS. Absorbance was measured at 640nm and results depicted as % of pI:C treated cells (n=3).

*(B)* Nuclease degradation of native PO or modified PS ssON 35 in serum free RPMI media. DNase I was used at a ratio of 1U/µg DNA. Degradation was carried out at 37°C for 0-24 hours at a concentration of 10µM ssON 35 PO/PS. Gel picture represents one out of three separate experiments.

*(C)* Cell viability: Flow cytometry of moDC 48h post initial treatment with 25μg/ml pI:C/0.5μM ssON 35 PS. Viable cells were assessed using a Live/Dead fixable near-IR dead cell stain kit according to manufacturer’s protocol (Life Technologies). n = >25 donors. Sample data was acquired using a Fortessa (BD Biosciences) and analyzed using FlowJo software (TreeStar Inc.).

*(D, E)* MoDCs were treated with ssON 35 PS at given concentrations for 4h *(D)* or 24h *(E)* and then exposed to WST-1 for 3h according to the manufacturer's protocol (Sigma). Absorbance was measured at 450nm. Untreated cells were defined as 100% viable. n = 2 donors.

Error bars are given in SEM. Non-parametric Mann-Whitney test was used to compare the data. P-value: not significant (n.s) P > 0.05; * P ≤ 0.05; ** P ≤ 0.01; *** P ≤ 0.001.


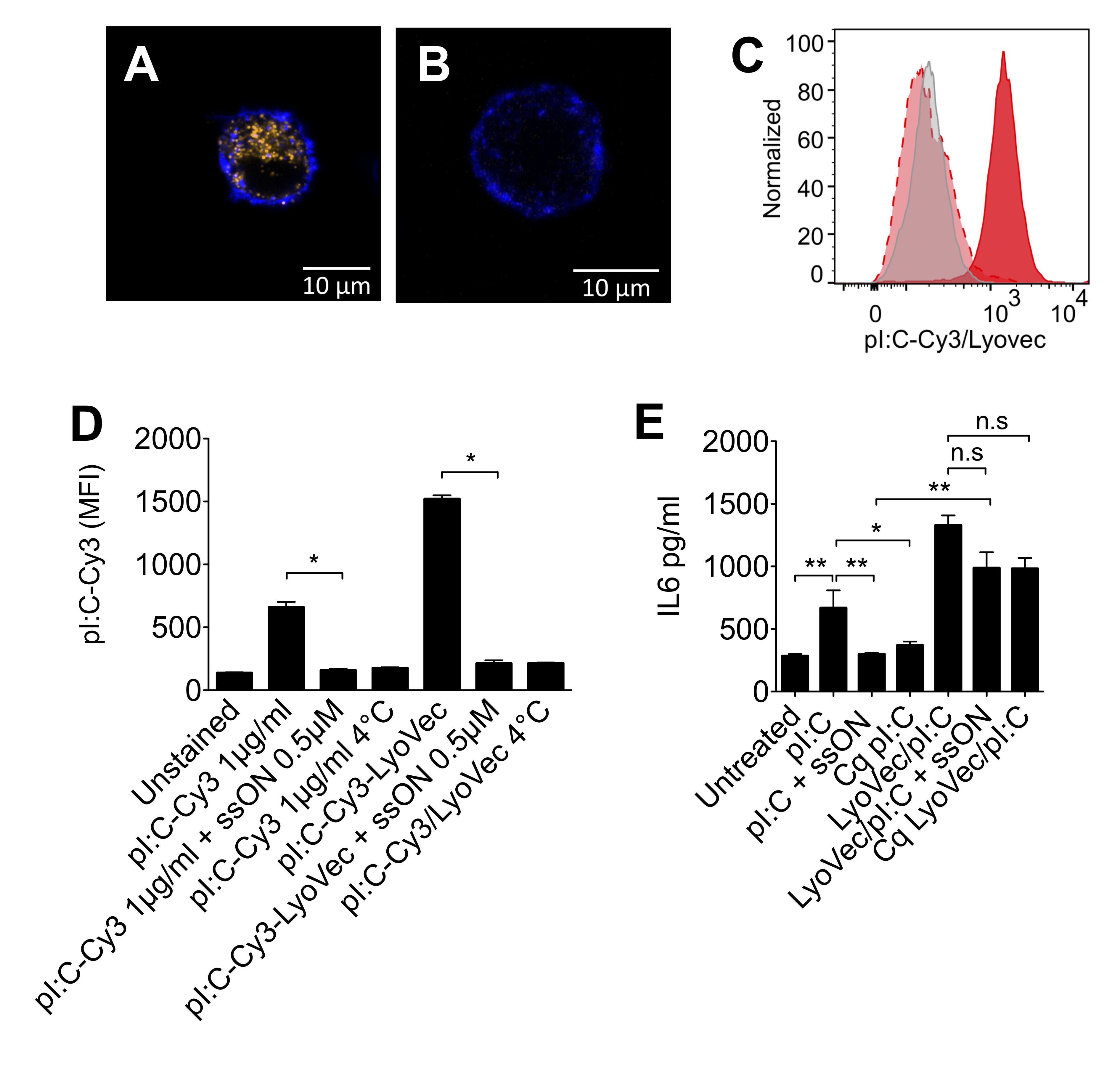


**S Fig 2.** SsON inhibit initial uptake of transfected pI:C, but not activation of cytosolic PRR.

Human moDC were treated for 45 min at +37^o^C or +4^o^C with pI:C-Cy3/LyoVec with or without addition of ssON. Flow cytometry histogram show representative data from at least two donors, in separate experiments. Red histogram is without ssON. Lighter color with dashed line depict the addition of ssON. Grey display background (fluorescent signal at +4°C).

*(A, B)* Confocal microscopy of pI:C-Cy3/LyoVec uptake in the absence *(A)* or presence of 0.5µM ssON 35 PS at +37^o^C *(B)*.

*(C)* Flow cytometry analysis of pI:C-Cy3/LyoVec uptake in the presence of 0.5µM ssON 35 PS at +37^o^C or +4^o^C.

(*D*) Quantification of pI:C-Cy3 and pI:C-Cy3/LyoVec uptake in the presence of 0.5µM ssON 35 PS at +37^o^C or +4^o^C.

(*E*) Both ssON 35 PS and pretreatment with chloroquine completely inhibited pI:C-induced IL-6 secretion, but not pI:C/LyoVec-induced IL-6 secretion in moDC (24h). Data from at least three donors, in separate experiments


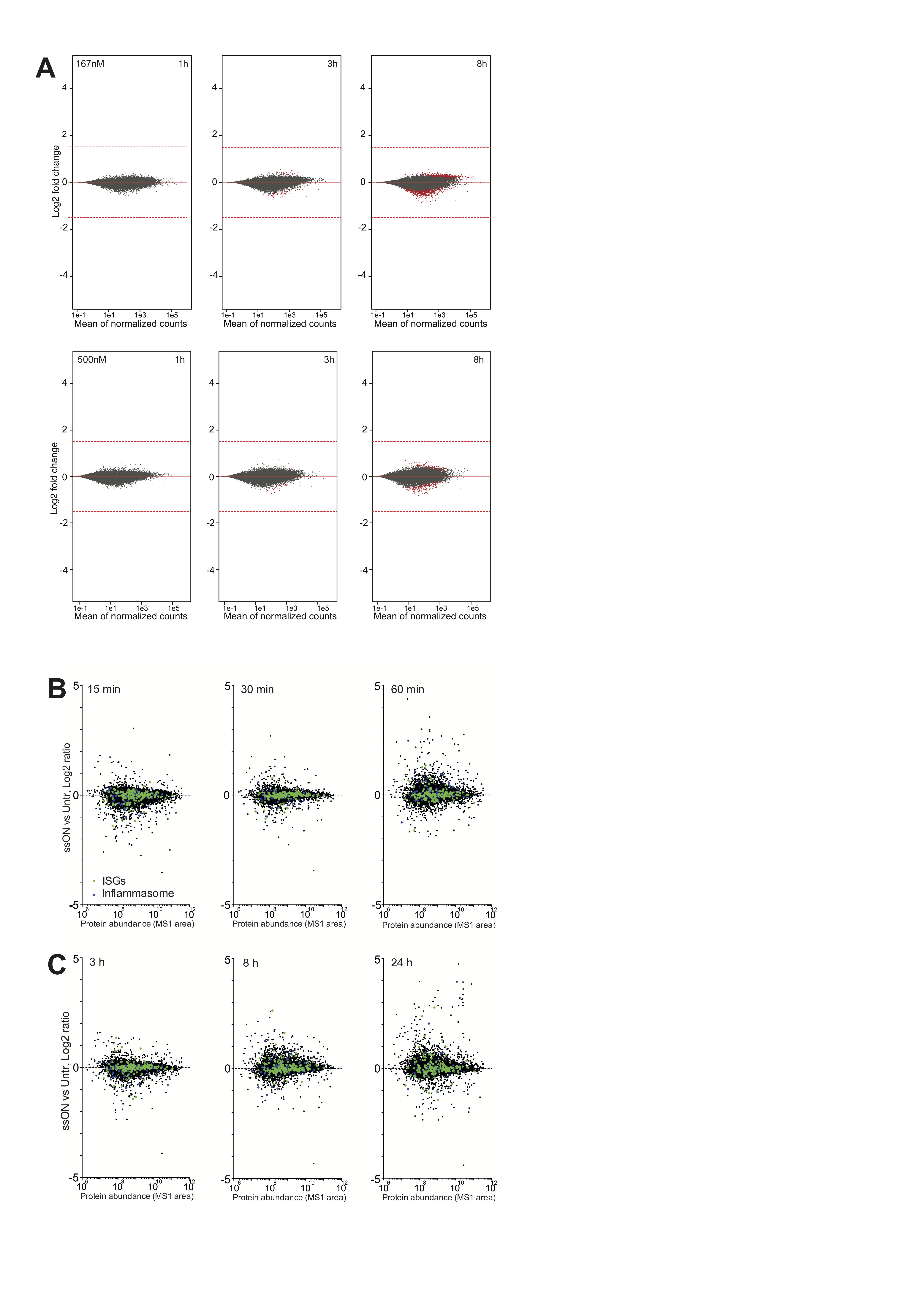


**S Fig 3. related to Fig 6.** No major transcriptomal changes detected after ssON exposure.

MA-plots of RNAseq data obtained from moDC treated with ssON for 1h, 3h or 8h using either *(A)* 167nM or *(B)* 500nM ssON 35 PS. MA-plots produced from the DESeq2 result after shrinkage of log_2_ fold changes (DESeq2 function lfcshrink) prior to visualization. Adjusted p-values below 0.05 are marked red.

*(C)* MA-plots of whole cell proteomic data (protein log intensity ratio versus average intensity) illustrating the time course of overall proteome changes in moDCs post treatment with ssON 35 PS (500nM). IFN-stimulated gene (ISG) proteins are colored green, inflammasome proteins are colored blue.


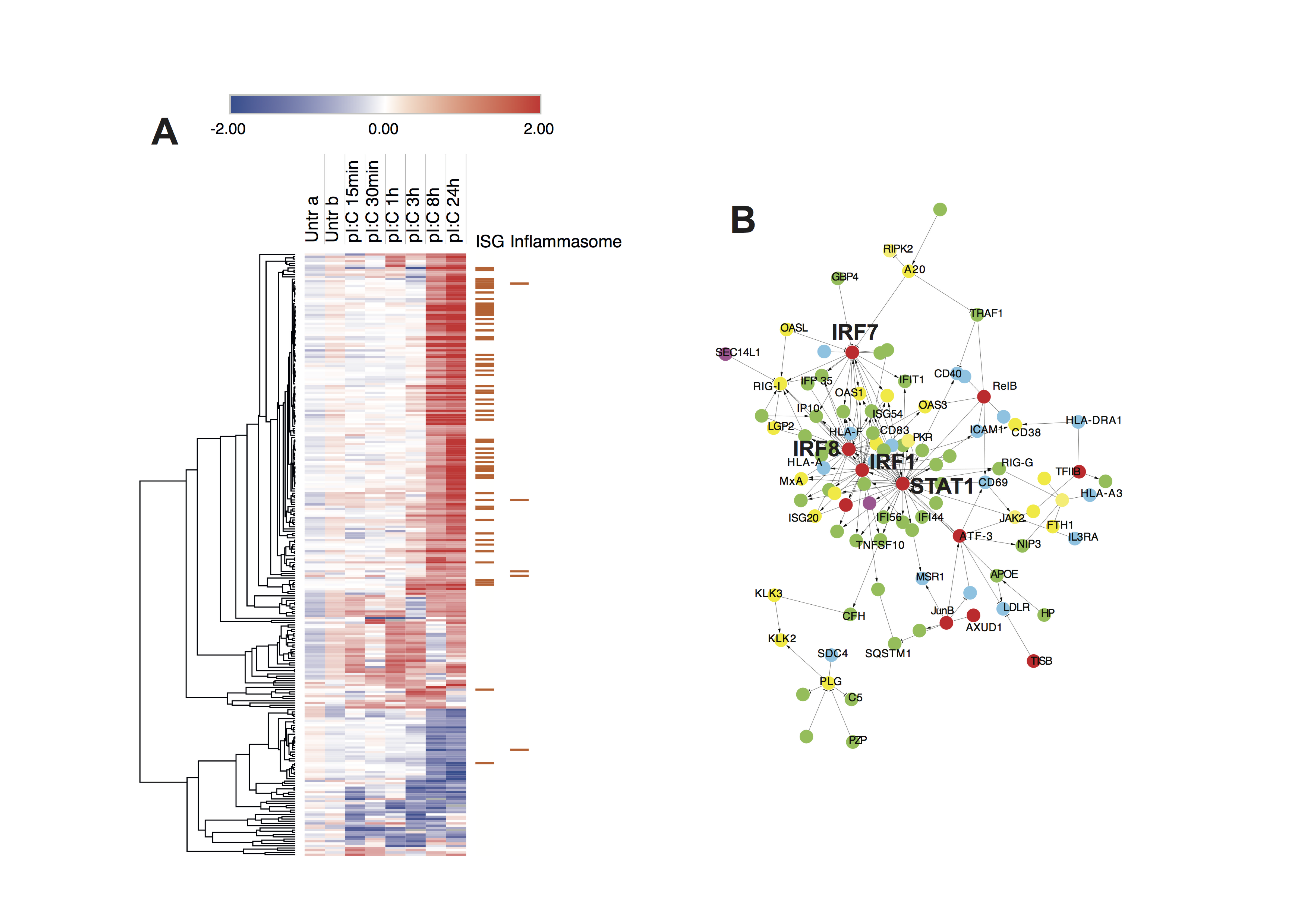


**S Fig 4. related to Fig 6D.** Differential protein expression in moDCs post-stimulation with pI:C.

*(A)* The heatmap shows up- or down regulated proteins, selected based on hierarchical clustering. Color indicates down-regulation (blue), up-regulation (red) or no change (white) relative the duplicate controls measured at time zero. ISG and Inflammasome related proteins are highlighted.

*(B)* Network analysis performed on the by pI:C up-regulated cluster showing the directly interconnected proteins. The four most interconnected proteins (>10 edges) were the transcription factors IRF-1, -7, -8 and STAT1 (bold). Network analysis performed on the other regulated clusters (down-regulated post pI:C stimulation, up- and down regulated based on ssON 35 PS stimulation) revealed no directly interconnected proteins. Network symbols indicate protein classes: red- transcription factor, blue – receptor, purple– transporter, green – ligand/binding protein, yellow - enzyme.


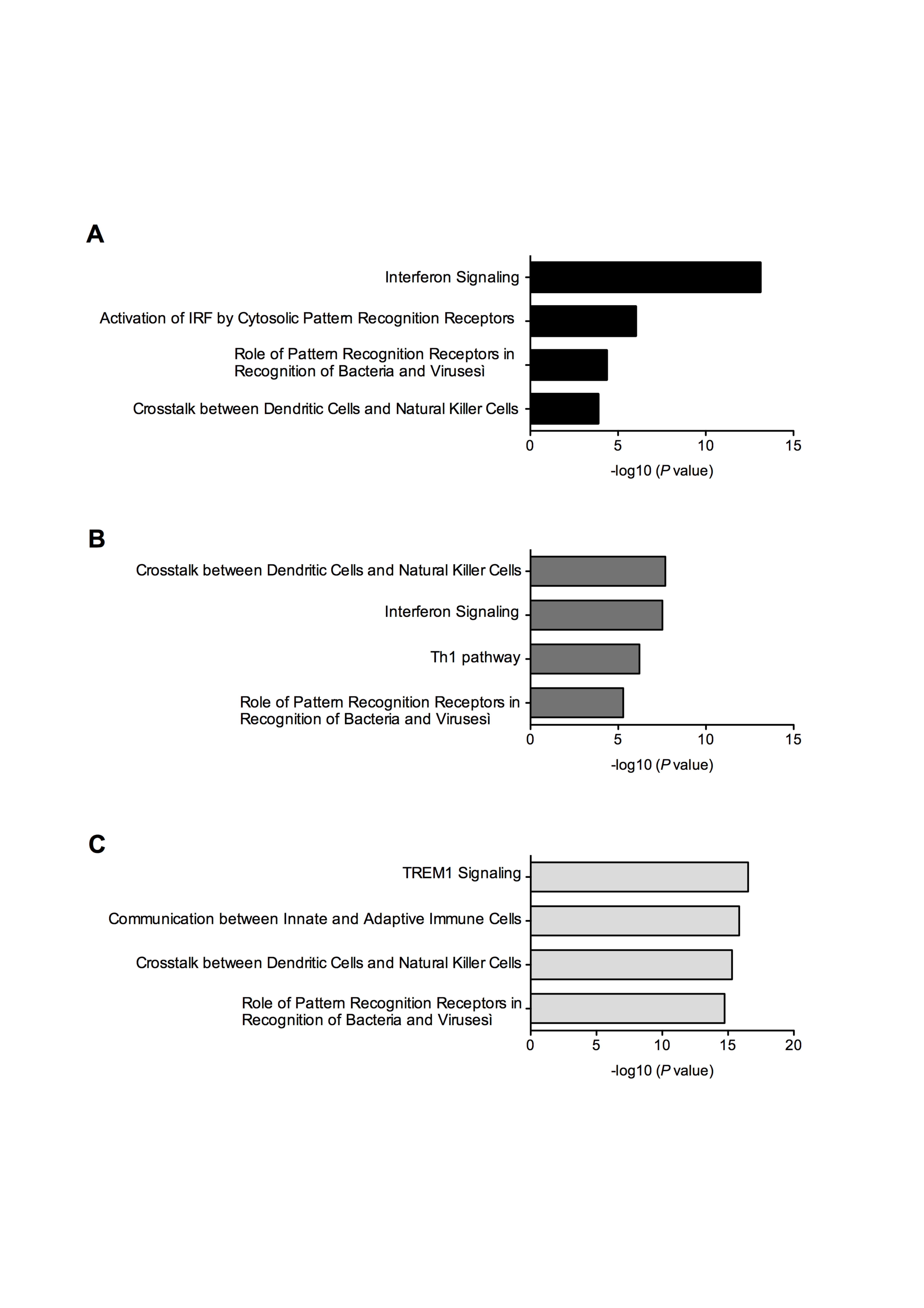


**S Fig 5. related to Fig 6 and 7.** Selected pathways significantly affected by pI:C stimulation.

The *P*-values associated with the pathway annotations were calculated using the right- tailed Fisher’s Exact Test, and corrected with the Benjamini-Hochberg method. A –log10(*P*-value) above 3 corresponds to a *P*-value <0.001.

*(A)* IPA analyses of RNAseq data from pI:C stimulated moDCs 24h post stimulation .

*(B)* IPA analyses of whole cell proteomic data from pI:C stimulated moDCs 24h post stimulation.

*(C)* IPA analyses of microarray data from pI:C stimulated macaque skin biopsies 24h post stimulation.


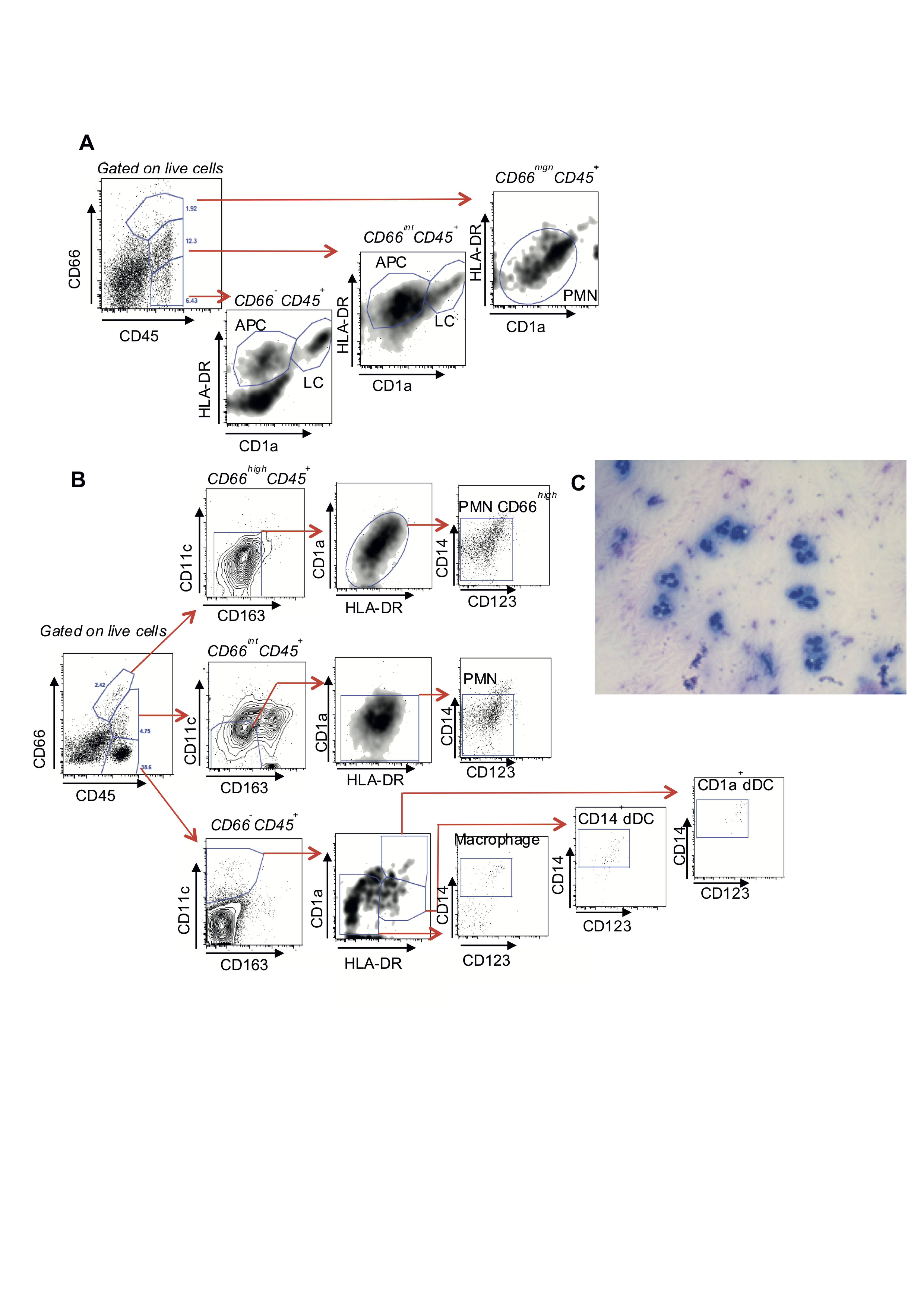


**S Fig 6. related to Fig 7.** Flow cytometry gating strategy.

Cells determined by size (FSC) and granularity (SSC), after selection of single cell events and exclusion of dead cells by viability staining. Three main gates were delineated for expression of CD66 and CD45. In the  epidermis *(A)*, LCs were identified as HLA-DR+CD1a+ and APCs by being HLA-DR+CD1a-. PMNs were identified as CD66high CD45+ cells (HLA-DR low-int, CD1-). In the dermis *(B),* cells in the three gates CD66- CD45+, CD66int CD45+ and CD66high CD45+ were further analyzed for CD11c and CD163 expression, followed by CD1a and HLA-DR expression. To allow further phenotypic characterization, expression of CD14 and CD123 were used to define five dermal cell populations: PMN CD66high,

PMN CD66int, Macrophages, CD14+ and CD1a+ dermal DC.

*(C)* Giemsa staining on dermal CD66high CD45+ FACS-sorted cells.


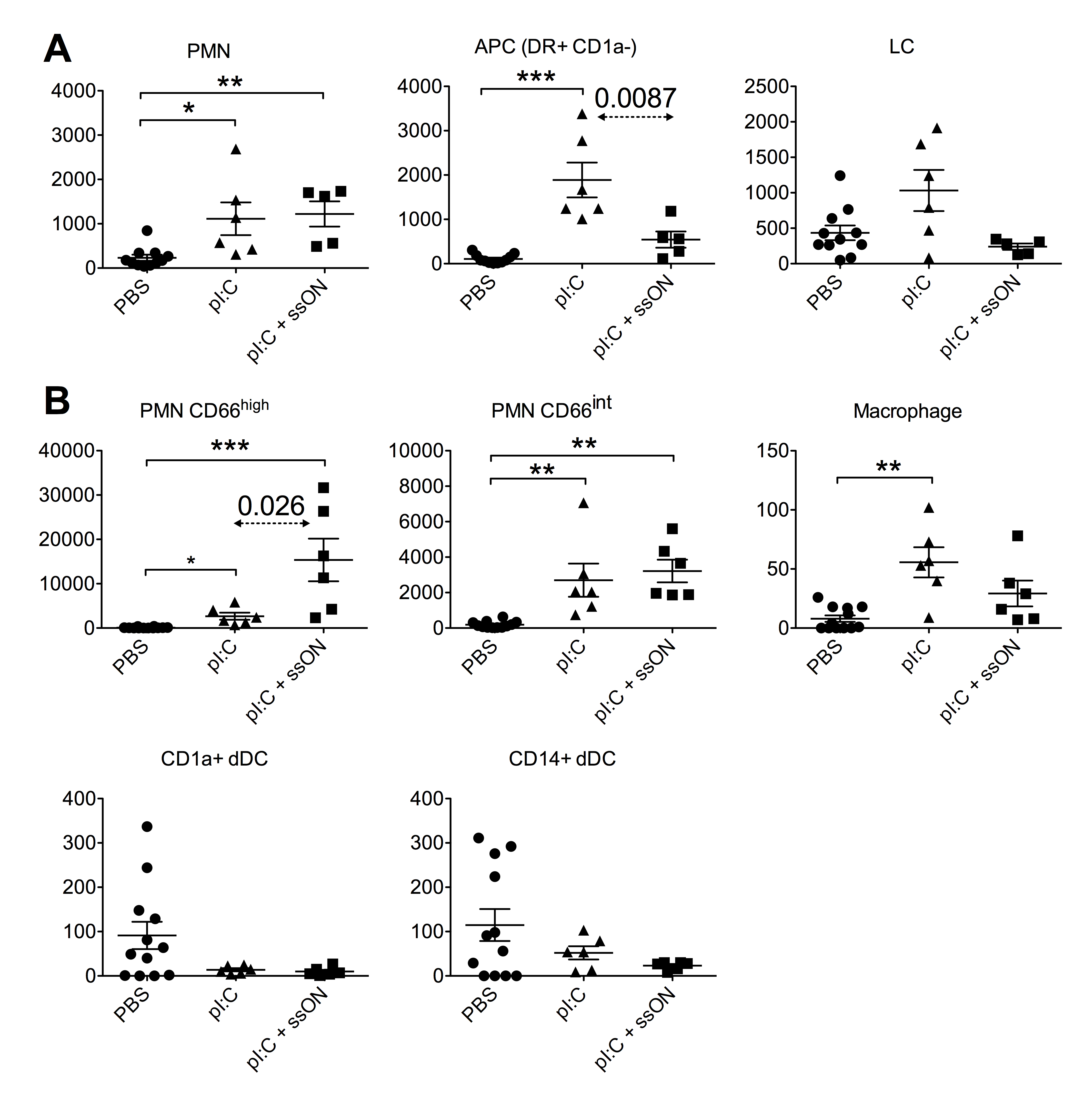


**S Fig 7 related to Fig 7.** Intradermal injection of dsRNA induces local inflammation in non-human primates.

The numbers of immune cells were identified by flow cytometry (see Figure S5 for gating strategy) in *(A)* epidermis and *(B)* dermis. Cells were collected from biopsies 24h after injection with either PBS (n=12), pI:C (n=6) or pI:C plus ssON (n=6). One outlier animal was excluded from results depicted from epidermis. Data are shown with means ± SEM. Significant differences were assessed by nonparametric Kruskal-Wallis test and Dunn's post-test (*P<0.05, ***P*<0.01 and ****P*<0.001).

**Supplementary Information, Material and Methods to:**

**Single-Stranded Nucleic Acids Regulate TLR3/4/7 Activation through Interference with Clathrin-Mediated Endocytosis**

**Detailed method: RNA sequencing and differential expression analysis**

Total RNA was purified from moDC using RNeasy total RNA purification kit, according to manufacturer’s instructions (Qiagen) and submitted to the National Genomics Infrastructure Sweden Stockholm (NGI) for sequencing. Prior to submission, bioanalyzer traces and concentration values were obtained following the guidelines in the *Sample requirements for genomics* documentation available at the NGI website. The RNA sequencing was performed with the TruSeq RiboZero kit from Illumina, 25 M reads per sample and 2x125bp. Fastq files were obtained from NGI and the read quality was assessed using FastQC (Version 0.11.5) (www.bioinformatics.babraham.ac.uk/projects/fastqc/). Trim Galore! (Version 0.3.6) (www.bioinformatics.babraham.ac.uk/projects/trim_galore) was used for adapter removal and quality trimming with a quality threshold of 20 on the Phred scale. Obtained reads were mapped to Homo sapiens UCSC hg38 (GRCh38.77) reference genome using STAR aligner (version 2.5)^1^ with default parameters. Count data for the aligned reads was generated with HTSeq-count (version 0.6.1)^2^ with the -m parameter set to union. The R/Bioconductor package DESeq2 (version 1.16.1)^3^ was used to conduct differential expression (DE) analysis according to the workflow outlined in the vignette. In the DESeq2 pipeline, the design formulae were set to investigate the condition parameter (i.e. treatment), while controlling for donor. The blind parameter was set to FALSE due to the expectation of differences between conditions. For each comparison, filtering was performed prior to analysis by removing all genes that had zero counts across samples. The DESeq2 function *lfcShrink* was used on all RNA-seq data to perform log2 fold change shrinkage for visualization and ranking of genes. Genes with adjusted p-value below 0.05 after Benjamini-Hochberg adjustment were labeled as differently expressed.

**Detailed method: Mass spectrometry based proteomics analysis of moDC**

For the kinetic *in vitro* study on human moDC, 2x8 samples were collected (time-points: 0h, 0h, 15min, 30min, 60min, 3h, 8h, 24h) after stimulation with either pI:C (25µg/ml) or ssON 35 PS (0.5µM). In separate experiments, moDC were stimulated with either pI:C (25µg/ml), ssON 35 PS (0.5µM), pI:C (25µg/ml) and ssON 35 PS (0.5µM) or incubated with medium for 24h. Cells were quickly spun and snap frozen. After cell lysis and protein extraction in 4% SDS, total protein amount was estimated (Bio-Rad). Protein digestion (trypsin, sequencing grade modified, Promega) to peptides was performed using a modified filter aided sample digestion protocol (FASP)^4^. 200µg of each protein sample was reduced, alkylated and digested (enzyme:protein ratio 1:50) in a centrifugation filtering unit with a 10kDa cut-off (Nanosep® Centrifugal Devices with Omega™ Membrane, 10k). For relative quantification, the peptides were labelled with isobaric tags (8-plex iTRAQ reagents (kinetic study), AB Sciex or 10-plex TMT reagents, Thermo Fisher Scientific (ssON+pI:C combination study) according to the manufacturer´s protocol, and then pooled.

To increase proteome coverage by reducing sample complexity, the samples (240µg per iTRAQ set, 350µg per TMT set) were subjected to high resolution peptide IEF-IPG (isoelectric focusing by immobilized pH gradient) in pI range 3.7–4.9^5-7^. After focusing, the peptides were passively eluted into 72 contiguous fractions using an in-house constructed IPG extractor robotics (GE Healthcare Bio- Sciences AB, prototype instrument) into a 96-well plate (V-bottom, Corning), and freeze-dried.

Liquid chromatography tandem mass spectrometry (LC-MS/MS) analysis was performed on each fraction using an Agilent 1200 nano-LC system coupled online to a Q Exactive Orbitrap (Thermo Fischer Scientific). The software Proteome Discoverer vs. 1.4.0.288 including Sequest-Percolator for improved identification^8^ was used to search the data against the human Ensembl database (version 37) for protein identification, limited to a false discovery rate of <1%.

In the kinetic study in total 8058 proteins (corresponding to 49 378 peptides) were detected in the whole cell lysates at a false discovery rate of 1%, with 6207 proteins detected in both pI:C and ssON time-series. For downstream cluster analyses, we defined protein expression as altered based on their log_2_ fold change in expression levels relative to the duplicate controls (95% confidence). Proteins for which the duplicate controls were dissimilar (>95% confidence) were considered uncertain and hence removed from analysis. Further, an increased stringency was applied by limiting the analysis to proteins for which the expression was altered in at least two consecutive time-points. In the ssON+pI:C combination study, a total of 8440 proteins (48 602 peptides) were detected at 1% FDR.

**Detailed method: NHP tissue collection and flow cytometry**

Cells were extracted from fresh skin biopsies (8mm in diameter) collected from anesthetized animals 24h after injection. The subcutaneous fat was removed and the biopsies collected for cell suspension analyses were incubated in PBS containing 4mg/ml grade II dispase (Roche Diagnostic) and 100μg/ml of Penicillin/Streptomycin/Neomycin (Life Technologies) over night at 4°C and then for 1h at 37°C with 5% CO_2_. Epidermis and dermis layers were separated, dermis was cut into small pieces, and layers were incubated for 20 or 40min, respectively, at 37°C with shaking in RPMI-1640 (Life Technologies) containing 2mg/ml of collagenase D, 0.02mg/mL DNAse I (both from Roche Diagnostic), 10mM HEPES (Life Technologies), 5% fetal calf serum (Lonza) and 100μg/ml of Penicillin/Streptomycin/Neomycin (Life Technologies). Cell suspensions were then filtered through a 70μm pore size filter. The residues on the filter were discarded for the epidermis while the dermal residues were mechanically dissociated through GentleMACS^TM^ dissociator (Miltenyi) and then re-filtered. Filtrates were centrifuged at 1800rpm for 10min before incubation with LIVE/DEAD Fixable Blue Dead Cell Stain Kit (Life Technologies), according to the manufacturer’s instructions. Epidermal and dermal cells were stained with a mix of monoclonal antibodies (HLA-DR-V500, CD123-PECy7, CD45-V450, CD11c-APC, CD14-APC-H7 from BD Bioscience; CD66-APC, CD66-FITC from Miltenyi; CD1a from DAKO; CD163-PcPCy5.5 from Biolegend) and acquired on a Fortessa flow cytometer (BD Biosciences). Data were analyzed with FlowJo software (Tree Star, version 9.6.4).

**Detailed method: Microarray analysis**

Whole skin RNA was extracted from macaque skin biopsies, stored at least 24h at 4°C in RNA Later, using Tissue Ruptor^®^ followed by RNeasy Plus Universal Kit (QIAgen), according to manufacturer’s instructions. Total RNA was quality checked on Agilent 2100 Bioanalyzer. RNA quantity was measured using NanoDrop ND-1000 Spectrophotometer (NanoDrop). Cyanine-3 (Cy3) labeled cRNA was prepared from 200ng Total RNA using the Quick Amp Labeling Kit (Agilent) according to the manufacturer's instructions, followed by RNeasy column purification (QIAGEN). Dye incorporation and cRNA yield were checked with the NanoDrop ND-1000 Spectrophotometer. 1.65µg of Cy3-labelled cRNA was fragmented at 60°C for 30min in a reaction volume of 55µL containing 1x Agilent fragmentation buffer and 2x Agilent blocking agent following the manufacturer’s instructions. On completion of the fragmentation reaction, 55µL of 2x Agilent hybridization buffer was added to the fragmentation mixture and hybridized to Agilent Rhesus Macaque Gene Expression Microarrays v2 for 17h at 65°C in a rotating Agilent hybridization oven. After hybridization, microarrays were washed 1min at room temperature with GE Wash Buffer 1 (Agilent) and 1min with 37°C GE Wash buffer 2 (Agilent). Slides were scanned immediately after washing on the Agilent DNA Microarray Scanner (G2505C) using one color scan setting for 4x44K array slides (Scan Area 61x21.6 mm, Scan resolution 5µm, Dye channel is set to Green, PMT is set to 100%). The scanned images were analyzed with Feature Extraction Software 10.7.3.1 (Agilent) using default parameters to obtain a background adjusted signal (gProcessedSignal) for each gene. The signals were subsequently quantile normalized to increase inter-sample comparability.

1. Dobin, A. *et al.* STAR: ultrafast universal RNA-seq aligner. *Bioinformatics* **29,** 15–21 (2013).

2. Anders, S., Pyl, P. T. & Huber, W. HTSeq--a Python framework to work with high-throughput sequencing data. *Bioinformatics* **31,** 166–169 (2015).

3. Love, M. I., Huber, W. & Anders, S. Moderated estimation of fold change and dispersion for RNA-seq data with DESeq2. *Genome Biol.* **15,** 550 (2014).

4. Hultin-Rosenberg, L., Forshed, J., Branca, R. M. M., Lehtiö, J. & Johansson, H. J. Defining, comparing, and improving iTRAQ quantification in mass spectrometry proteomics data. *Mol. Cell Proteomics* **12,** 2021–2031 (2013).

5. Eriksson, H. *et al.* Quantitative membrane proteomics applying narrow range peptide isoelectric focusing for studies of small cell lung cancer resistance mechanisms. *Proteomics* **8,** 3008–3018 (2008).

6. Lengqvist, J., Uhlén, K. & Lehtiö, J. iTRAQ compatibility of peptide immobilized pH gradient isoelectric focusing. *Proteomics* **7,** 1746–1752 (2007).

7. Sandberg, A., Branca, R. M. M., Lehtiö, J. & Forshed, J. Quantitative accuracy in mass spectrometry based proteomics of complex samples: the impact of labeling and precursor interference. *J Proteomics* **96,** 133–144 (2014).

8. Käll, L., Canterbury, J. D., Weston, J., Noble, W. S. & MacCoss, M. J. Semi-supervised learning for peptide identification from shotgun proteomics datasets. *Nat. Methods* **4,** 923–925 (2007).
